# Supplementary material for: Catalytic Reaction Mechanism of NO–CO on the ZrO2 (110) and (111) Surfaces
Source: Int J Mol Sci. 2019 Dec 5;20(24):6129. doi: 10.3390/ijms20246129 (PMC6940978; doi:10.3390/ijms20246129)
Supplement: Supplementary file 1 [file ijms-20-06129-s001.pdf]

Supplementary information for

“Catalytic reaction mechanism of NO-CO on the ZrO_2 (110) and (111) surfaces”

Xuesong Cao[1], Chenxi Zhang[2,*], Zehua Wang[1] and Xiaomin Sun[1,*]

Supporting information list:

Table S1. Surface formation energies of ZrO_2 (100), (110) and (111) surface.

Table S2. The bond distances for NO, CO, N_2 and CO_2 molecules.

Table S3. Cartesian coordinates for all the optimized structures of reactants, transition states and products

Surface formation energies (E_{surf}) were calculated by the following formula:

$$E_{surf} = (E_{slab} - nE_{bulk})/2A_{slab} \quad (2)$$

where E_{slab} is the energy of surface, E_{bulk} is the energy of optimized bulk, A_{slab} is the area of surface and n is the number of atoms in the optimized bulk cell. Surface energies of ZrO_2 (100), (110) and (111) were given on the Table S1 below.

Table S1. Surface formation energies of ZrO_2 (100), (110) and (111) surface.

ZrO_2 Surfaces	(100)	(110)	(111)
Surface energy (kcal.mol^{-1}. Å^{-2})	7.016	2.753	1.487

Table S2. The bond distances for NO, CO, N_2 and CO_2 molecules.

Molecule	NO	CO	N_2	CO_2
Calculated value (Å)	1.163	1.140	1.107	1.175
B3LYP/6-31G(d) value (Å)	1.159	1.138	1.106	1.169

Table S3. Cartesian coordinates for all the optimized structures of reactants, transition states and products

CO C-end adsorbed on Zr_T site of ZrO_2 (110) surface

ATOM		X (Angstroms)	Y (Angstroms)	Z (Angstroms)
1	O	1.361327	-0.000270	3.607460
2	O	1.311525	0.000000	0.000000
3	O	4.012751	1.955519	5.471802
4	O	3.934575	1.854776	1.854776
5	O	3.937650	-0.000169	3.727643
6	O	3.934575	0.000000	0.000000
7	O	1.280127	1.728707	5.486759
8	O	1.311525	1.854776	1.854776
9	Zr	0.000000	0.000000	1.854776
10	Zr	2.623050	1.854776	0.000000
11	Zr	2.631289	1.862048	3.831980
12	Zr	0.134161	-0.000158	5.238795
13	O	6.597789	-0.000033	3.606974
14	O	6.557625	0.000000	0.000000
15	O	9.249503	1.981520	5.491926
16	O	9.180675	1.854776	1.854776
17	O	9.192646	-0.000177	3.748218
18	O	9.180675	0.000000	0.000000
19	O	6.533577	1.712742	5.491770
20	O	6.557625	1.854776	1.854776
21	Zr	5.246100	0.000000	1.854776
22	Zr	7.869150	1.854776	0.000000
23	Zr	7.872090	1.854125	3.844423
24	Zr	5.350467	0.000018	5.231501
25	O	1.279214	3.709521	3.732507
26	O	1.311525	3.709553	0.000000
27	O	4.012548	5.462976	5.471922
28	O	3.934575	5.564329	1.854776
29	O	3.855261	3.709728	3.621334
30	O	3.934575	3.709553	0.000000
31	O	1.279920	5.690326	5.486235
32	O	1.311525	5.564329	1.854776
33	Zr	0.000000	3.709553	1.854776
34	Zr	2.623050	5.564329	0.000000
35	Zr	2.630709	5.556848	3.831914
36	Zr	-0.114318	3.709429	5.241925
37	O	6.532773	3.709676	3.747795
38	O	6.557625	3.709553	0.000000
39	O	9.249223	5.437151	5.491742
40	O	9.180675	5.564329	1.854776
41	O	9.111796	3.709390	3.619194
42	O	9.180675	3.709553	0.000000
43	O	6.533317	5.705912	5.491447
44	O	6.557625	5.564329	1.854776
45	Zr	5.246100	3.709553	1.854776
46	Zr	7.869150	5.564329	0.000000
47	Zr	7.872366	5.564679	3.844072
48	Zr	5.142294	3.709786	5.265345
49	C	5.860902	3.708933	7.762439
50	O	6.283094	3.711159	8.818711

CO C-end adsorbed on O_T site of ZrO_2 (110) surface

ATOM		X (Angstroms)	Y (Angstroms)	Z (Angstroms)
1	O	1.365528	0.003025	3.609630
2	O	1.311525	0.000000	0.000000
3	O	4.024130	1.956337	5.458994
4	O	3.934575	1.854776	1.854776
5	O	3.947534	-0.003084	3.724679
6	O	3.934575	0.000000	0.000000

7	O	1.281900	1.733582	5.487898
8	O	1.311525	1.854776	1.854776
9	Zr	0.000000	0.000000	1.854776
10	Zr	2.623050	1.854776	0.000000
11	Zr	2.642006	1.866671	3.840300
12	Zr	0.133506	0.007364	5.241091
13	O	6.612352	-0.003266	3.608897
14	O	6.557625	0.000000	0.000000
15	O	9.252045	1.982125	5.493272
16	O	9.180675	1.854776	1.854776
17	O	9.198935	0.001214	3.745483
18	O	9.180675	0.000000	0.000000
19	O	6.545496	1.688976	5.504901
20	O	6.557625	1.854776	1.854776
21	Zr	5.246100	0.000000	1.854776
22	Zr	7.869150	1.854776	0.000000
23	Zr	7.870443	1.845677	3.846365
24	Zr	5.354377	-0.002435	5.229652
25	O	1.280695	3.715646	3.733912
26	O	1.311525	3.709553	0.000000
27	O	3.999219	5.470991	5.485416
28	O	3.934575	5.564329	1.854776
29	O	3.857417	3.723382	3.622711
30	O	3.934575	3.709553	0.000000
31	O	1.271806	5.697780	5.486002
32	O	1.311525	5.564329	1.854776
33	Zr	0.000000	3.709553	1.854776
34	Zr	2.623050	5.564329	0.000000
35	Zr	2.617955	5.564735	3.822500
36	Zr	-0.117341	3.711225	5.241998
37	O	6.533536	3.698773	3.745100
38	O	6.557625	3.709553	0.000000
39	O	9.240203	5.436225	5.484033
40	O	9.180675	5.564329	1.854776
41	O	9.115774	3.705483	3.614916
42	O	9.180675	3.709553	0.000000
43	O	6.513596	5.675309	5.472736
44	O	6.557625	5.564329	1.854776
45	Zr	5.246100	3.709553	1.854776
46	Zr	7.869150	5.564329	0.000000
47	Zr	7.876109	5.560346	3.834484
48	Zr	5.143070	3.750602	5.264151
49	C	5.758243	3.321233	7.734956
50	O	6.088230	2.978535	8.769077

CO C-end adsorbed on O_b site of ZrO_2 (110) surface

ATOM		X (Angstroms)	Y (Angstroms)	Z (Angstroms)
1	O	1.370202	-0.002664	3.616256
2	O	1.311525	0.000000	0.000000
3	O	3.977563	1.989138	5.494469
4	O	3.934575	1.854776	1.854776
5	O	3.956147	0.000239	3.747050
6	O	3.934575	0.000000	0.000000
7	O	1.262121	1.723938	5.486469
8	O	1.311525	1.854776	1.854776
9	Zr	0.000000	0.000000	1.854776
10	Zr	2.623050	1.854776	0.000000
11	Zr	2.621039	1.853634	3.841982
12	Zr	0.115113	0.006511	5.241784
13	O	6.619184	-0.001677	3.610402
14	O	6.557625	0.000000	0.000000
15	O	9.236686	1.978726	5.480144
16	O	9.180675	1.854776	1.854776
17	O	9.201460	0.000881	3.738259
18	O	9.180675	0.000000	0.000000
19	O	6.499804	1.732197	5.478629
20	O	6.557625	1.854776	1.854776
21	Zr	5.246100	0.000000	1.854776

22	Zr	7.869150	1.854776	0.000000
23	Zr	7.868553	1.856506	3.833168
24	Zr	5.372070	-0.006254	5.244003
25	O	1.289157	3.709780	3.741403
26	O	1.311525	3.709553	0.000000
27	O	3.982868	5.436473	5.494534
28	O	3.934575	5.564329	1.854776
29	O	3.874350	3.711342	3.615348
30	O	3.934575	3.709553	0.000000
31	O	1.255258	5.687642	5.487901
32	O	1.311525	5.564329	1.854776
33	Zr	0.000000	3.709553	1.854776
34	Zr	2.623050	5.564329	0.000000
35	Zr	2.620569	5.565170	3.846831
36	Zr	-0.122700	3.718283	5.241295
37	O	6.538165	3.710281	3.740275
38	O	6.557625	3.709553	0.000000
39	O	9.228294	5.435566	5.481072
40	O	9.180675	5.564329	1.854776
41	O	9.119606	3.711729	3.609509
42	O	9.180675	3.709553	0.000000
43	O	6.507515	5.695310	5.482392
44	O	6.557625	5.564329	1.854776
45	Zr	5.246100	3.709553	1.854776
46	Zr	7.869150	5.564329	0.000000
47	Zr	7.868032	5.563735	3.827924
48	Zr	5.122928	3.703623	5.242738
49	C	7.841967	1.466690	9.758415
50	O	7.877722	2.047772	10.739212

CO O-end adsorbed on Zr_T site of ZrO_2 (110) surface

ATOM		X (Angstroms)	Y (Angstroms)	Z (Angstroms)
1	O	1.383450	0.001459	3.615975
2	O	1.311525	0.000000	0.000000
3	O	3.991942	1.972725	5.474840
4	O	3.934575	1.854776	1.854776
5	O	3.964958	0.000657	3.729749
6	O	3.934575	0.000000	0.000000
7	O	1.261782	1.733354	5.489185
8	O	1.311525	1.854776	1.854776
9	Zr	0.000000	0.000000	1.854776
10	Zr	2.623050	1.854776	0.000000
11	Zr	2.632171	1.858111	3.833374
12	Zr	0.130628	0.001135	5.242013
13	O	6.628740	0.000414	3.609465
14	O	6.557625	0.000000	0.000000
15	O	9.235649	1.981405	5.488082
16	O	9.180675	1.854776	1.854776
17	O	9.211282	0.000442	3.740677
18	O	9.180675	0.000000	0.000000
19	O	6.510923	1.725188	5.485368
20	O	6.557625	1.854776	1.854776
21	Zr	5.246100	0.000000	1.854776
22	Zr	7.869150	1.854776	0.000000
23	Zr	7.876475	1.854265	3.840498
24	Zr	5.371037	0.001171	5.232908
25	O	1.301131	3.709064	3.740953
26	O	1.311525	3.709553	0.000000
27	O	3.991433	5.447312	5.475306
28	O	3.934575	5.564329	1.854776
29	O	3.883366	3.709994	3.615921
30	O	3.934575	3.709553	0.000000
31	O	1.261798	5.686872	5.487043
32	O	1.311525	5.564329	1.854776
33	Zr	0.000000	3.709553	1.854776
34	Zr	2.623050	5.564329	0.000000
35	Zr	2.631591	5.560944	3.833131
36	Zr	-0.111705	3.709153	5.241420

37	O	6.544569	3.710095	3.747252
38	O	6.557625	3.709553	0.000000
39	O	9.234676	5.436305	5.486255
40	O	9.180675	5.564329	1.854776
41	O	9.129220	3.707866	3.614323
42	O	9.180675	3.709553	0.000000
43	O	6.509259	5.691959	5.484401
44	O	6.557625	5.564329	1.854776
45	Zr	5.246100	3.709553	1.854776
46	Zr	7.869150	5.564329	0.000000
47	Zr	7.875092	5.563607	3.839439
48	Zr	5.137168	3.710820	5.253976
49	C	5.419294	3.652738	9.129701
50	O	5.377578	3.671677	7.989204

CO O-end adsorbed on O_T site of ZrO_2 (110) surface

ATOM		X (Angstroms)	Y (Angstroms)	Z (Angstroms)
1	O	1.369469	-0.002964	3.612850
2	O	1.311525	0.000000	0.000000
3	O	3.983945	1.981758	5.491436
4	O	3.934575	1.854776	1.854776
5	O	3.955163	0.003791	3.740196
6	O	3.934575	0.000000	0.000000
7	O	1.260588	1.729615	5.481838
8	O	1.311525	1.854776	1.854776
9	Zr	0.000000	0.000000	1.854776
10	Zr	2.623050	1.854776	0.000000
11	Zr	2.624999	1.856148	3.838942
12	Zr	0.119287	0.001666	5.240750
13	O	6.617697	-0.005157	3.611008
14	O	6.557625	0.000000	0.000000
15	O	9.232732	1.980477	5.491787
16	O	9.180675	1.854776	1.854776
17	O	9.201388	0.006155	3.739372
18	O	9.180675	0.000000	0.000000
19	O	6.503858	1.727607	5.475682
20	O	6.557625	1.854776	1.854776
21	Zr	5.246100	0.000000	1.854776
22	Zr	7.869150	1.854776	0.000000
23	Zr	7.869884	1.853567	3.833528
24	Zr	5.366444	-0.001386	5.241791
25	O	1.289623	3.713362	3.740917
26	O	1.311525	3.709553	0.000000
27	O	3.985054	5.439956	5.483157
28	O	3.934575	5.564329	1.854776
29	O	3.873270	3.706802	3.617297
30	O	3.934575	3.709553	0.000000
31	O	1.259786	5.693922	5.489726
32	O	1.311525	5.564329	1.854776
33	Zr	0.000000	3.709553	1.854776
34	Zr	2.623050	5.564329	0.000000
35	Zr	2.619879	5.564284	3.839503
36	Zr	-0.123560	3.708985	5.241226
37	O	6.537461	3.714983	3.744800
38	O	6.557625	3.709553	0.000000
39	O	9.231910	5.437589	5.477359
40	O	9.180675	5.564329	1.854776
41	O	9.118355	3.703369	3.612485
42	O	9.180675	3.709553	0.000000
43	O	6.507541	5.694709	5.490321
44	O	6.557625	5.564329	1.854776
45	Zr	5.246100	3.709553	1.854776
46	Zr	7.869150	5.564329	0.000000
47	Zr	7.862984	5.562726	3.834174
48	Zr	5.124599	3.706569	5.246813
49	O	6.087746	2.154099	8.928528
50	C	6.641573	1.890831	9.889521

CO O-end adsorbed on O_b site of ZrO_2 (110) surface

ATOM		X (Angstroms)	Y (Angstroms)	Z (Angstroms)
1	O	1.369464	0.001597	3.615882
2	O	1.311525	0.000000	0.000000
3	O	3.981254	1.985764	5.492460
4	O	3.934575	1.854776	1.854776
5	O	3.954986	0.002380	3.743362
6	O	3.934575	0.000000	0.000000
7	O	1.261586	1.724869	5.492173
8	O	1.311525	1.854776	1.854776
9	Zr	0.000000	0.000000	1.854776
10	Zr	2.623050	1.854776	0.000000
11	Zr	2.621783	1.854674	3.844102
12	Zr	0.114669	0.003684	5.241227
13	O	6.617696	0.000561	3.612863
14	O	6.557625	0.000000	0.000000
15	O	9.235151	1.973854	5.478917
16	O	9.180675	1.854776	1.854776
17	O	9.199423	-0.002385	3.737704
18	O	9.180675	0.000000	0.000000
19	O	6.501318	1.732959	5.481444
20	O	6.557625	1.854776	1.854776
21	Zr	5.246100	0.000000	1.854776
22	Zr	7.869150	1.854776	0.000000
23	Zr	7.866571	1.855317	3.832256
24	Zr	5.367790	-0.001344	5.243596
25	O	1.289056	3.707218	3.743369
26	O	1.311525	3.709553	0.000000
27	O	3.984543	5.439046	5.484065
28	O	3.934575	5.564329	1.854776
29	O	3.873875	3.707787	3.615563
30	O	3.934575	3.709553	0.000000
31	O	1.255657	5.687412	5.483447
32	O	1.311525	5.564329	1.854776
33	Zr	0.000000	3.709553	1.854776
34	Zr	2.623050	5.564329	0.000000
35	Zr	2.621371	5.564462	3.840675
36	Zr	-0.124123	3.712319	5.242405
37	O	6.537475	3.711526	3.741905
38	O	6.557625	3.709553	0.000000
39	O	9.227365	5.436613	5.485916
40	O	9.180675	5.564329	1.854776
41	O	9.117700	3.709867	3.613230
42	O	9.180675	3.709553	0.000000
43	O	6.506537	5.694510	5.487124
44	O	6.557625	5.564329	1.854776
45	Zr	5.246100	3.709553	1.854776
46	Zr	7.869150	5.564329	0.000000
47	Zr	7.866707	5.563761	3.832288
48	Zr	5.124272	3.706981	5.242673
49	O	7.870067	1.844290	8.816789
50	C	7.869809	1.844955	9.956443

NO N-end adsorbed on Zr_T site of ZrO_2 (110) surface

ATOM		X (Angstroms)	Y (Angstroms)	Z (Angstroms)
1	O	1.373840	-0.000029	3.610459
2	O	1.311525	0.000000	0.000000
3	O	4.011269	1.939121	5.457375
4	O	3.934575	1.854776	1.854776
5	O	3.961628	0.000052	3.705624
6	O	3.934575	0.000000	0.000000
7	O	1.274524	1.736792	5.483745
8	O	1.311525	1.854776	1.854776
9	Zr	0.000000	0.000000	1.854776
10	Zr	2.623050	1.854776	0.000000
11	Zr	2.642724	1.864242	3.852563
12	Zr	0.134883	0.000097	5.239331

13	O	6.618732	-0.000069	3.593583
14	O	6.557625	0.000000	0.000000
15	O	9.249557	1.979889	5.482846
16	O	9.180675	1.854776	1.854776
17	O	9.214867	0.000086	3.737515
18	O	9.180675	0.000000	0.000000
19	O	6.532295	1.704063	5.474180
20	O	6.557625	1.854776	1.854776
21	Zr	5.246100	0.000000	1.854776
22	Zr	7.869150	1.854776	0.000000
23	Zr	7.869026	1.857798	3.864373
24	Zr	5.360662	0.000321	5.227801
25	O	1.289905	3.709677	3.727087
26	O	1.311525	3.709553	0.000000
27	O	4.010367	5.479919	5.457344
28	O	3.934575	5.564329	1.854776
29	O	3.874289	3.709631	3.633860
30	O	3.934575	3.709553	0.000000
31	O	1.273950	5.682351	5.483785
32	O	1.311525	5.564329	1.854776
33	Zr	0.000000	3.709553	1.854776
34	Zr	2.623050	5.564329	0.000000
35	Zr	2.642327	5.555029	3.852199
36	Zr	-0.106747	3.709730	5.242172
37	O	6.546773	3.709511	3.759946
38	O	6.557625	3.709553	0.000000
39	O	9.248965	5.439385	5.482753
40	O	9.180675	5.564329	1.854776
41	O	9.129685	3.709556	3.611492
42	O	9.180675	3.709553	0.000000
43	O	6.531271	5.714131	5.473867
44	O	6.557625	5.564329	1.854776
45	Zr	5.246100	3.709553	1.854776
46	Zr	7.869150	5.564329	0.000000
47	Zr	7.869007	5.560951	3.864256
48	Zr	5.150713	3.710319	5.309301
49	N	5.514821	3.712616	7.674010
50	O	5.084797	3.719381	8.773214

NO N-end adsorbed on O_T site of ZrO_2 (110) surface

ATOM		X (Angstroms)	Y (Angstroms)	Z (Angstroms)
1	O	1.368414	-0.000687	3.608378
2	O	1.311525	0.000000	0.000000
3	O	4.021929	1.939502	5.455258
4	O	3.934575	1.854776	1.854776
5	O	3.957082	0.002874	3.708888
6	O	3.934575	0.000000	0.000000
7	O	1.281772	1.736468	5.483507
8	O	1.311525	1.854776	1.854776
9	Zr	0.000000	0.000000	1.854776
10	Zr	2.623050	1.854776	0.000000
11	Zr	2.650653	1.864499	3.857452
12	Zr	0.134133	0.002124	5.237887
13	O	6.614819	-0.003122	3.596258
14	O	6.557625	0.000000	0.000000
15	O	9.256103	1.977520	5.483109
16	O	9.180675	1.854776	1.854776
17	O	9.207064	0.001857	3.737254
18	O	9.180675	0.000000	0.000000
19	O	6.538426	1.707849	5.470500
20	O	6.557625	1.854776	1.854776
21	Zr	5.246100	0.000000	1.854776
22	Zr	7.869150	1.854776	0.000000
23	Zr	7.868581	1.854901	3.861710
24	Zr	5.358981	0.004212	5.229693
25	O	1.283918	3.711707	3.725406
26	O	1.311525	3.709553	0.000000
27	O	4.013894	5.484094	5.449832

28	O	3.934575	5.564329	1.854776
29	O	3.869821	3.709342	3.632667
30	O	3.934575	3.709553	0.000000
31	O	1.278007	5.684001	5.487263
32	O	1.311525	5.564329	1.854776
33	Zr	0.000000	3.709553	1.854776
34	Zr	2.623050	5.564329	0.000000
35	Zr	2.636493	5.555414	3.848566
36	Zr	-0.104516	3.710759	5.242585
37	O	6.538565	3.708986	3.750904
38	O	6.557625	3.709553	0.000000
39	O	9.253384	5.445556	5.479762
40	O	9.180675	5.564329	1.854776
41	O	9.126953	3.709635	3.614922
42	O	9.180675	3.709553	0.000000
43	O	6.531355	5.701609	5.474859
44	O	6.557625	5.564329	1.854776
45	Zr	5.246100	3.709553	1.854776
46	Zr	7.869150	5.564329	0.000000
47	Zr	7.872134	5.562863	3.860300
48	Zr	5.160876	3.728227	5.307226
49	N	5.408160	3.660190	7.691687
50	O	5.713407	3.033855	8.644285

NO N-end adsorbed on O_b site of ZrO_2 (110) surface

ATOM		X (Angstroms)	Y (Angstroms)	Z (Angstroms)
1	O	1.368247	0.001106	3.618106
2	O	1.311525	0.000000	0.000000
3	O	3.979069	1.986069	5.495766
4	O	3.934575	1.854776	1.854776
5	O	3.957078	0.002531	3.745482
6	O	3.934575	0.000000	0.000000
7	O	1.261886	1.725229	5.491879
8	O	1.311525	1.854776	1.854776
9	Zr	0.000000	0.000000	1.854776
10	Zr	2.623050	1.854776	0.000000
11	Zr	2.621658	1.854479	3.854758
12	Zr	0.110358	0.005494	5.243161
13	O	6.619106	-0.000527	3.610520
14	O	6.557625	0.000000	0.000000
15	O	9.237968	1.970738	5.468997
16	O	9.180675	1.854776	1.854776
17	O	9.200993	-0.002573	3.733608
18	O	9.180675	0.000000	0.000000
19	O	6.499074	1.736558	5.469940
20	O	6.557625	1.854776	1.854776
21	Zr	5.246100	0.000000	1.854776
22	Zr	7.869150	1.854776	0.000000
23	Zr	7.867027	1.855784	3.833763
24	Zr	5.371202	-0.001716	5.246609
25	O	1.287371	3.706980	3.743140
26	O	1.311525	3.709553	0.000000
27	O	3.984201	5.439663	5.487843
28	O	3.934575	5.564329	1.854776
29	O	3.875472	3.708057	3.618778
30	O	3.934575	3.709553	0.000000
31	O	1.254800	5.685924	5.483934
32	O	1.311525	5.564329	1.854776
33	Zr	0.000000	3.709553	1.854776
34	Zr	2.623050	5.564329	0.000000
35	Zr	2.621228	5.564436	3.853100
36	Zr	-0.125237	3.712950	5.244710
37	O	6.537020	3.712559	3.738638
38	O	6.557625	3.709553	0.000000
39	O	9.228535	5.438148	5.479327
40	O	9.180675	5.564329	1.854776
41	O	9.117253	3.710562	3.610676
42	O	9.180675	3.709553	0.000000

43	O	6.506872	5.693854	5.481657
44	O	6.557625	5.564329	1.854776
45	Zr	5.246100	3.709553	1.854776
46	Zr	7.869150	5.564329	0.000000
47	Zr	7.867060	5.563715	3.833393
48	Zr	5.128374	3.704977	5.246750
49	N	7.818828	1.781721	8.891890
50	O	7.910188	1.891170	10.055146

NO O-end adsorbed on Zr_T site of ZrO_2 (110) surface

ATOM		X (Angstroms)	Y (Angstroms)	Z (Angstroms)
1	O	1.369005	0.000883	3.613104
2	O	1.311525	0.000000	0.000000
3	O	4.004418	1.948451	5.457866
4	O	3.934575	1.854776	1.854776
5	O	3.959779	-0.002183	3.716988
6	O	3.934575	0.000000	0.000000
7	O	1.270015	1.732069	5.489144
8	O	1.311525	1.854776	1.854776
9	Zr	0.000000	0.000000	1.854776
10	Zr	2.623050	1.854776	0.000000
11	Zr	2.639189	1.859745	3.854567
12	Zr	0.128584	0.000666	5.242642
13	O	6.614487	-0.000828	3.604512
14	O	6.557625	0.000000	0.000000
15	O	9.243859	1.979205	5.487999
16	O	9.180675	1.854776	1.854776
17	O	9.206612	0.000637	3.739980
18	O	9.180675	0.000000	0.000000
19	O	6.522219	1.721989	5.468043
20	O	6.557625	1.854776	1.854776
21	Zr	5.246100	0.000000	1.854776
22	Zr	7.869150	1.854776	0.000000
23	Zr	7.865993	1.856471	3.861529
24	Zr	5.358625	0.001246	5.238757
25	O	1.285965	3.708030	3.734134
26	O	1.311525	3.709553	0.000000
27	O	4.004595	5.469985	5.466197
28	O	3.934575	5.564329	1.854776
29	O	3.872612	3.710363	3.631099
30	O	3.934575	3.709553	0.000000
31	O	1.270257	5.683767	5.484609
32	O	1.311525	5.564329	1.854776
33	Zr	0.000000	3.709553	1.854776
34	Zr	2.623050	5.564329	0.000000
35	Zr	2.635959	5.556984	3.851857
36	Zr	-0.112113	3.709724	5.244440
37	O	6.533418	3.713400	3.751183
38	O	6.557625	3.709553	0.000000
39	O	9.244235	5.443078	5.481742
40	O	9.180675	5.564329	1.854776
41	O	9.123033	3.707949	3.615345
42	O	9.180675	3.709553	0.000000
43	O	6.521302	5.700569	5.478125
44	O	6.557625	5.564329	1.854776
45	Zr	5.246100	3.709553	1.854776
46	Zr	7.869150	5.564329	0.000000
47	Zr	7.868270	5.566128	3.860780
48	Zr	5.148189	3.714877	5.291001
49	O	5.406689	3.617336	7.755632
50	N	5.445624	2.654082	8.459674

NO O-end adsorbed on O_T site of ZrO_2 (110) surface

ATOM		X (Angstroms)	Y (Angstroms)	Z (Angstroms)
1	O	1.372964	-0.004191	3.612634
2	O	1.311525	0.000000	0.000000
3	O	3.986206	1.976706	5.484539

4	O	3.934575	1.854776	1.854776
5	O	3.959868	0.001783	3.739694
6	O	3.934575	0.000000	0.000000
7	O	1.259975	1.732231	5.474072
8	O	1.311525	1.854776	1.854776
9	Zr	0.000000	0.000000	1.854776
10	Zr	2.623050	1.854776	0.000000
11	Zr	2.628627	1.855005	3.841461
12	Zr	0.122265	0.002199	5.242500
13	O	6.618111	-0.003468	3.608917
14	O	6.557625	0.000000	0.000000
15	O	9.235092	1.979999	5.485005
16	O	9.180675	1.854776	1.854776
17	O	9.205434	0.003825	3.737126
18	O	9.180675	0.000000	0.000000
19	O	6.505422	1.728701	5.469927
20	O	6.557625	1.854776	1.854776
21	Zr	5.246100	0.000000	1.854776
22	Zr	7.869150	1.854776	0.000000
23	Zr	7.873259	1.853189	3.837481
24	Zr	5.369810	0.000917	5.245542
25	O	1.290253	3.713902	3.736894
26	O	1.311525	3.709553	0.000000
27	O	3.990907	5.439102	5.485561
28	O	3.934575	5.564329	1.854776
29	O	3.877092	3.707471	3.617441
30	O	3.934575	3.709553	0.000000
31	O	1.263846	5.691011	5.485988
32	O	1.311525	5.564329	1.854776
33	Zr	0.000000	3.709553	1.854776
34	Zr	2.623050	5.564329	0.000000
35	Zr	2.626828	5.563105	3.846940
36	Zr	-0.118850	3.710918	5.242755
37	O	6.536580	3.712926	3.739909
38	O	6.557625	3.709553	0.000000
39	O	9.238106	5.439203	5.476445
40	O	9.180675	5.564329	1.854776
41	O	9.121797	3.707860	3.611362
42	O	9.180675	3.709553	0.000000
43	O	6.510623	5.694460	5.481316
44	O	6.557625	5.564329	1.854776
45	Zr	5.246100	3.709553	1.854776
46	Zr	7.869150	5.564329	0.000000
47	Zr	7.865916	5.563606	3.838900
48	Zr	5.130264	3.705275	5.251286
49	O	5.917233	2.029621	8.811008
50	N	6.582832	1.972488	9.773460

NO O-end adsorbed on O_b site of ZrO_2 (110) surface

ATOM		X (Angstroms)	Y (Angstroms)	Z (Angstroms)
1	O	1.369825	0.000546	3.615578
2	O	1.311525	0.000000	0.000000
3	O	3.982138	1.983650	5.490297
4	O	3.934575	1.854776	1.854776
5	O	3.956106	0.001353	3.742884
6	O	3.934575	0.000000	0.000000
7	O	1.261108	1.726504	5.488130
8	O	1.311525	1.854776	1.854776
9	Zr	0.000000	0.000000	1.854776
10	Zr	2.623050	1.854776	0.000000
11	Zr	2.622088	1.854697	3.845243
12	Zr	0.115693	0.003597	5.242442
13	O	6.618135	-0.000056	3.612061
14	O	6.557625	0.000000	0.000000
15	O	9.235643	1.975477	5.477991
16	O	9.180675	1.854776	1.854776
17	O	9.201317	-0.001363	3.737184
18	O	9.180675	0.000000	0.000000

19	O	6.502156	1.731799	5.479421
20	O	6.557625	1.854776	1.854776
21	Zr	5.246100	0.000000	1.854776
22	Zr	7.869150	1.854776	0.000000
23	Zr	7.867666	1.855413	3.834529
24	Zr	5.368527	-0.001581	5.244181
25	O	1.288456	3.708071	3.741727
26	O	1.311525	3.709553	0.000000
27	O	3.984271	5.438948	5.486444
28	O	3.934575	5.564329	1.854776
29	O	3.873963	3.708969	3.616105
30	O	3.934575	3.709553	0.000000
31	O	1.256921	5.688045	5.483747
32	O	1.311525	5.564329	1.854776
33	Zr	0.000000	3.709553	1.854776
34	Zr	2.623050	5.564329	0.000000
35	Zr	2.621741	5.564307	3.844442
36	Zr	-0.123034	3.712406	5.243499
37	O	6.537009	3.710744	3.740944
38	O	6.557625	3.709553	0.000000
39	O	9.229797	5.437533	5.482054
40	O	9.180675	5.564329	1.854776
41	O	9.118547	3.710208	3.612248
42	O	9.180675	3.709553	0.000000
43	O	6.506212	5.693506	5.483306
44	O	6.557625	5.564329	1.854776
45	Zr	5.246100	3.709553	1.854776
46	Zr	7.869150	5.564329	0.000000
47	Zr	7.867813	5.563887	3.834471
48	Zr	5.125454	3.706879	5.244918
49	O	7.842440	1.801465	8.719928
50	N	7.895087	1.882513	9.881962

Path 1--i

ATOM		X (Angstroms)	Y (Angstroms)	Z (Angstroms)
1	O	1.330192	-0.000015	3.688909
2	O	1.311525	0.000000	0.000000
3	O	4.001779	1.854778	5.480281
4	O	3.934575	1.854776	1.854776
5	O	3.915908	-0.000015	3.688909
6	O	3.934575	0.000000	0.000000
7	O	1.244321	1.854778	5.480281
8	O	1.311525	1.854776	1.854776
9	Zr	0.000000	0.000000	1.854776
10	Zr	2.623050	1.854776	0.000000
11	Zr	2.623050	1.854778	3.863164
12	Zr	0.000000	-0.000044	5.235956
13	O	6.576292	-0.000015	3.688909
14	O	6.557625	0.000000	0.000000
15	O	9.247879	1.854778	5.480281
16	O	9.180675	1.854776	1.854776
17	O	9.162008	-0.000015	3.688909
18	O	9.180675	0.000000	0.000000
19	O	6.490421	1.854778	5.480281
20	O	6.557625	1.854776	1.854776
21	Zr	5.246100	0.000000	1.854776
22	Zr	7.869150	1.854776	0.000000
23	Zr	7.869150	1.854778	3.863164
24	Zr	5.246100	-0.000044	5.235956
25	O	1.330192	3.709570	3.688908
26	O	1.311525	3.709553	0.000000
27	O	4.001786	5.564328	5.480287
28	O	3.934575	5.564329	1.854776
29	O	3.915908	3.709570	3.688908
30	O	3.934575	3.709553	0.000000
31	O	1.244314	5.564328	5.480287
32	O	1.311525	5.564329	1.854776
33	Zr	0.000000	3.709553	1.854776

34	Zr	2.623050	5.564329	0.000000
35	Zr	2.623050	5.564328	3.863042
36	Zr	0.000000	3.709597	5.235955
37	O	6.576292	3.709570	3.688908
38	O	6.557625	3.709553	0.000000
39	O	9.247886	5.564328	5.480287
40	O	9.180675	5.564329	1.854776
41	O	9.162008	3.709570	3.688908
42	O	9.180675	3.709553	0.000000
43	O	6.490414	5.564328	5.480287
44	O	6.557625	5.564329	1.854776
45	Zr	5.246100	3.709553	1.854776
46	Zr	7.869150	5.564329	0.000000
47	Zr	7.869150	5.564328	3.863042
48	Zr	5.246100	3.709597	5.235955

Path 1--ii

ATOM		X (Angstroms)	Y (Angstroms)	Z (Angstroms)
1	O	1.359791	-0.000114	3.608218
2	O	1.311525	0.000000	0.000000
3	O	4.013780	1.954811	5.471044
4	O	3.934575	1.854776	1.854776
5	O	3.939782	-0.000077	3.727951
6	O	3.934575	0.000000	0.000000
7	O	1.278824	1.728148	5.486503
8	O	1.311525	1.854776	1.854776
9	Zr	0.000000	0.000000	1.854776
10	Zr	2.623050	1.854776	0.000000
11	Zr	2.630910	1.861198	3.831844
12	Zr	0.132181	-0.000008	5.239937
13	O	6.600625	0.000037	3.606289
14	O	6.557625	0.000000	0.000000
15	O	9.248675	1.981984	5.491161
16	O	9.180675	1.854776	1.854776
17	O	9.192229	-0.000102	3.747853
18	O	9.180675	0.000000	0.000000
19	O	6.533070	1.714298	5.491124
20	O	6.557625	1.854776	1.854776
21	Zr	5.246100	0.000000	1.854776
22	Zr	7.869150	1.854776	0.000000
23	Zr	7.872059	1.854314	3.844011
24	Zr	5.353272	0.000116	5.231175
25	O	1.279409	3.709585	3.732827
26	O	1.311525	3.709553	0.000000
27	O	4.013479	5.464036	5.471389
28	O	3.934575	5.564329	1.854776
29	O	3.855089	3.709698	3.621537
30	O	3.934575	3.709553	0.000000
31	O	1.278668	5.690973	5.486333
32	O	1.311525	5.564329	1.854776
33	Zr	0.000000	3.709553	1.854776
34	Zr	2.623050	5.564329	0.000000
35	Zr	2.630385	5.557837	3.831821
36	Zr	-0.114357	3.709478	5.242408
37	O	6.532164	3.709649	3.747669
38	O	6.557625	3.709553	0.000000
39	O	9.248532	5.436925	5.491091
40	O	9.180675	5.564329	1.854776
41	O	9.112218	3.709476	3.618689
42	O	9.180675	3.709553	0.000000
43	O	6.532793	5.704595	5.490992
44	O	6.557625	5.564329	1.854776
45	Zr	5.246100	3.709553	1.854776
46	Zr	7.869150	5.564329	0.000000
47	Zr	7.872268	5.564607	3.843769
48	Zr	5.141393	3.709924	5.265560
49	C	5.856057	3.709756	7.762034
50	O	6.285300	3.710808	8.816583

Path 1—TS1

ATOM	X (au)	Y (au)	Z (au)
O	2.48065647803592	0.00080786736707	6.77794368086571
O	2.47842305608222	0.00000000000000	0.00000000000000
O	8.99917119031007	3.35582686245188	10.75365725292114
O	7.43526916824665	3.50501949847006	3.50501949847006
O	7.32412226547077	0.03113434717850	7.01962187181928
O	7.43526916824665	0.00000000000000	0.00000000000000
O	2.50576737043416	3.32451576540945	10.27519814351007
O	2.47842305608222	3.50501949847006	3.50501949847006
Zr	0.00000000000000	0.00000000000000	3.50501949847006
Zr	4.95684611216444	3.50501949847006	0.00000000000000
Zr	4.89561612088984	3.63043852756934	6.84514444324593
Zr	0.26864247193315	0.03978162999154	9.85911766516307
O	12.45734268494693	0.30157360472637	7.17286412478290
O	12.39211528041109	0.00000000000000	0.00000000000000
O	17.34743008324575	3.67167735183202	10.20655752509590
O	17.34896139257553	3.50501949847006	3.50501949847006
O	17.40554170327094	-0.02843248479513	6.96308969433799
O	17.34896139257553	0.00000000000000	0.00000000000000
O	12.03089210205271	6.51872284670956	13.92921363687720
O	12.39211528041109	3.50501949847006	3.50501949847006
Zr	9.91369222432887	0.00000000000000	3.50501949847006
Zr	14.87053833649331	3.50501949847006	0.00000000000000
Zr	15.06454856955832	3.52586907546397	6.74812504794282
Zr	9.75474062308203	-0.33177745643010	10.04119571583032
O	2.33707301213962	6.99772026539432	6.98159776649248
O	2.47842305608222	7.01003899882984	0.00000000000000
O	7.55660875485305	10.45687585380803	10.43750760477760
O	7.43526916824665	10.51505849729990	3.50501949847006
O	7.20130708244022	6.88551283130955	7.06431719602692
O	7.43526916824665	7.01003899882984	0.00000000000000
O	2.37882278622116	10.70910634725315	10.29728404547538
O	2.47842305608222	10.51505849729990	3.50501949847006
Zr	0.00000000000000	7.01003899882984	3.50501949847006
Zr	4.95684611216444	10.51505849729990	0.00000000000000
Zr	4.97043043298039	10.37534508468292	7.25054213162273
Zr	-0.24402604905222	6.95382558791022	9.92244270319587
O	12.47526012320106	6.70403675667886	7.40888141814137
O	12.39211528041109	7.01003899882984	0.00000000000000
O	17.49034767390629	10.27479347377822	10.29794424019440
O	17.34896139257553	10.51505849729990	3.50501949847006
O	17.30808992729208	7.03516788897970	6.76723835213200
O	17.34896139257553	7.01003899882984	0.00000000000000
O	12.38962080224185	10.69607942194475	10.26591794039518
O	12.39211528041109	10.51505849729990	3.50501949847006
Zr	9.91369222432887	7.01003899882984	3.50501949847006
Zr	14.87053833649331	10.51505849729990	0.00000000000000
Zr	14.89297624908227	10.49919581495326	7.32271457257547
Zr	10.16426243841565	7.20836081023465	10.75906088887076
C	11.92417197343148	9.08468231980786	15.40553188160838
O	13.72123379051606	9.56886510351591	16.69091854814999

Path 1--iii

ATOM		X (Angstroms)	Y (Angstroms)	Z (Angstroms)
1	O	1.339528	-0.005987	3.580705
2	O	1.311525	0.000000	0.000000
3	O	5.208322	1.838425	5.919675
4	O	3.934575	1.854776	1.854776
5	O	3.870944	0.115626	3.800654
6	O	3.934575	0.000000	0.000000
7	O	1.329610	1.782346	5.455317
8	O	1.311525	1.854776	1.854776
9	Zr	0.000000	0.000000	1.854776
10	Zr	2.623050	1.854776	0.000000
11	Zr	2.507246	1.863043	3.543151
12	Zr	0.099511	0.061280	5.234873

13	O	6.625870	0.119714	3.803996
14	O	6.557625	0.000000	0.000000
15	O	9.158123	1.928960	5.453664
16	O	9.180675	1.854776	1.854776
17	O	9.203029	-0.019770	3.674366
18	O	9.180675	0.000000	0.000000
19	O	6.076511	4.065781	7.993011
20	O	6.557625	1.854776	1.854776
21	Zr	5.246100	0.000000	1.854776
22	Zr	7.869150	1.854776	0.000000
23	Zr	7.986811	1.846361	3.546420
24	Zr	5.233435	-0.140933	5.306952
25	O	1.294867	3.719089	3.663409
26	O	1.311525	3.709553	0.000000
27	O	3.982131	5.556671	5.539890
28	O	3.934575	5.564329	1.854776
29	O	3.874921	3.595939	3.821165
30	O	3.934575	3.709553	0.000000
31	O	1.244669	5.665271	5.444041
32	O	1.311525	5.564329	1.854776
33	Zr	0.000000	3.709553	1.854776
34	Zr	2.623050	5.564329	0.000000
35	Zr	2.553915	5.560641	3.834414
36	Zr	-0.093651	3.651220	5.228603
37	O	6.612883	3.581394	3.840853
38	O	6.557625	3.709553	0.000000
39	O	9.232453	5.443603	5.469179
40	O	9.180675	5.564329	1.854776
41	O	9.151468	3.720732	3.577998
42	O	9.180675	3.709553	0.000000
43	O	6.511518	5.587890	5.532006
44	O	6.557625	5.564329	1.854776
45	Zr	5.246100	3.709553	1.854776
46	Zr	7.869150	5.564329	0.000000
47	Zr	7.942181	5.561975	3.846693
48	Zr	5.244616	3.849604	5.348789
49	C	6.811191	4.929199	8.322370
50	O	7.533122	5.765330	8.704014

Path 1--iv

ATOM		X (Angstroms)	Y (Angstroms)	Z (Angstroms)
1	O	1.307545	-0.015546	3.621478
2	O	1.311525	0.000000	0.000000
3	O	5.252680	1.854784	5.948561
4	O	3.934575	1.854776	1.854776
5	O	3.865726	0.119067	3.826313
6	O	3.934575	0.000000	0.000000
7	O	1.324553	1.854731	5.439124
8	O	1.311525	1.854776	1.854776
9	Zr	0.000000	0.000000	1.854776
10	Zr	2.623050	1.854776	0.000000
11	Zr	2.492142	1.854787	3.560287
12	Zr	-0.008968	0.046688	5.223328
13	O	6.612807	0.120312	3.815198
14	O	6.557625	0.000000	0.000000
15	O	9.166593	1.854817	5.437920
16	O	9.180675	1.854776	1.854776
17	O	9.170518	-0.012354	3.616026
18	O	9.180675	0.000000	0.000000
19	O	6.557625	1.854776	1.854776
20	Zr	5.246100	0.000000	1.854776
21	Zr	7.869150	1.854776	0.000000
22	Zr	7.988147	1.854774	3.548966
23	Zr	5.246538	-0.143786	5.328794
24	O	1.307513	3.725093	3.621524
25	O	1.311525	3.709553	0.000000
26	O	3.987732	5.564327	5.548626
27	O	3.934575	5.564329	1.854776

28	O	3.865710	3.590498	3.826310
29	O	3.934575	3.709553	0.000000
30	O	1.248013	5.564375	5.449872
31	O	1.311525	5.564329	1.854776
32	Zr	0.000000	3.709553	1.854776
33	Zr	2.623050	5.564329	0.000000
34	Zr	2.551610	5.564325	3.854178
35	Zr	-0.009081	3.662892	5.223326
36	O	6.612798	3.589242	3.815200
37	O	6.557625	3.709553	0.000000
38	O	9.252715	5.564266	5.436165
39	O	9.180675	5.564329	1.854776
40	O	9.170488	3.721906	3.615977
41	O	9.180675	3.709553	0.000000
42	O	6.510218	5.564335	5.541539
43	O	6.557625	5.564329	1.854776
44	Zr	5.246100	3.709553	1.854776
45	Zr	7.869150	5.564329	0.000000
46	Zr	7.931460	5.564332	3.832107
47	Zr	5.246541	3.853344	5.328796

Path 1--v

ATOM		X (Angstroms)	Y (Angstroms)	Z (Angstroms)
1	O	1.258977	0.021374	3.659740
2	O	1.311525	0.000000	0.000000
3	O	4.114932	1.865154	5.377422
4	O	3.934575	1.854776	1.854776
5	O	3.829547	-0.049815	3.676146
6	O	3.934575	0.000000	0.000000
7	O	1.303342	1.852590	5.495782
8	O	1.311525	1.854776	1.854776
9	Zr	0.000000	0.000000	1.854776
10	Zr	2.623050	1.854776	0.000000
11	Zr	2.635325	1.865343	3.869318
12	Zr	-0.020766	0.000131	5.225253
13	O	6.513862	0.038599	3.663191
14	O	6.557625	0.000000	0.000000
15	O	9.294506	1.868501	5.423108
16	O	9.180675	1.854776	1.854776
17	O	9.117286	-0.020729	3.679276
18	O	9.180675	0.000000	0.000000
19	O	6.557625	1.854776	1.854776
20	Zr	5.246100	0.000000	1.854776
21	Zr	7.869150	1.854776	0.000000
22	Zr	7.850215	1.837633	3.777220
23	Zr	5.194856	-0.068468	5.253769
24	O	1.249920	3.693596	3.663770
25	O	1.311525	3.709553	0.000000
26	O	3.973774	5.584949	5.609566
27	O	3.934575	5.564329	1.854776
28	O	3.823540	3.782241	3.691712
29	O	3.934575	3.709553	0.000000
30	O	1.252086	5.576250	5.433485
31	O	1.311525	5.564329	1.854776
32	Zr	0.000000	3.709553	1.854776
33	Zr	2.623050	5.564329	0.000000
34	Zr	2.563026	5.564387	3.793477
35	Zr	-0.042650	3.707202	5.225362
36	O	6.510363	3.647537	3.755798
37	O	6.557625	3.709553	0.000000
38	O	9.253697	5.550151	5.468259
39	O	9.180675	5.564329	1.854776
40	O	9.109473	3.722736	3.660224
41	O	9.180675	3.709553	0.000000
42	O	6.485854	5.616822	5.420574
43	O	6.557625	5.564329	1.854776
44	Zr	5.246100	3.709553	1.854776
45	Zr	7.869150	5.564329	0.000000

46	Zr	7.858118	5.544997	3.848997
47	Zr	5.151672	3.884391	5.336393
48	N	6.640776	1.590898	5.725858
49	O	6.463976	2.658746	6.509313

Path 1--vi

ATOM		X (Angstroms)	Y (Angstroms)	Z (Angstroms)
1	O	1.270769	0.024082	3.633660
2	O	1.311525	0.000000	0.000000
3	O	4.125909	1.857265	5.383993
4	O	3.934575	1.854776	1.854776
5	O	3.834605	-0.031722	3.655948
6	O	3.934575	0.000000	0.000000
7	O	1.311469	1.791746	5.496111
8	O	1.311525	1.854776	1.854776
9	Zr	0.000000	0.000000	1.854776
10	Zr	2.623050	1.854776	0.000000
11	Zr	2.633611	1.867160	3.859500
12	Zr	0.024212	0.012124	5.225008
13	O	6.521229	0.031600	3.652476
14	O	6.557625	0.000000	0.000000
15	O	9.295783	1.910336	5.422981
16	O	9.180675	1.854776	1.854776
17	O	9.128872	-0.024773	3.701280
18	O	9.180675	0.000000	0.000000
19	O	6.557625	1.854776	1.854776
20	Zr	5.246100	0.000000	1.854776
21	Zr	7.869150	1.854776	0.000000
22	Zr	7.853161	1.845509	3.765199
23	Zr	5.196508	-0.067392	5.234250
24	O	1.232999	3.698929	3.687247
25	O	1.311525	3.709553	0.000000
26	O	3.951096	5.623167	5.558210
27	O	3.934575	5.564329	1.854776
28	O	3.813167	3.777738	3.704009
29	O	3.934575	3.709553	0.000000
30	O	1.239525	5.625334	5.439775
31	O	1.311525	5.564329	1.854776
32	Zr	0.000000	3.709553	1.854776
33	Zr	2.623050	5.564329	0.000000
34	Zr	2.559394	5.570886	3.793275
35	Zr	-0.098419	3.719992	5.228758
36	O	6.495154	3.639721	3.769125
37	O	6.557625	3.709553	0.000000
38	O	9.235317	5.499398	5.474489
39	O	9.180675	5.564329	1.854776
40	O	9.087982	3.722947	3.632216
41	O	9.180675	3.709553	0.000000
42	O	6.471256	5.620577	5.434158
43	O	6.557625	5.564329	1.854776
44	Zr	5.246100	3.709553	1.854776
45	Zr	7.869150	5.564329	0.000000
46	Zr	7.845619	5.541663	3.848685
47	Zr	5.124764	3.893039	5.370567
48	N	6.668365	1.573432	5.715170
49	O	6.524698	2.640102	6.488916
50	C	4.262216	3.374275	7.729734
51	O	3.954256	2.861041	8.701276

Path 1—TS2

ATOM	X (au)	Y (au)	Z (au)
O	2.48076119531941	0.01556193810302	6.86046716091879
O	2.47842305608222	0.00000000000000	0.00000000000000
O	7.54298372744664	3.35527973193663	10.05752349946057
O	7.43526916824665	3.50501949847006	3.50501949847006
O	7.37450372834536	-0.12099813199288	6.86027167064089
O	7.43526916824665	0.00000000000000	0.00000000000000
O	2.35610879598370	3.36952250136468	10.38824228596202

O	2.47842305608222	3.50501949847006	3.50501949847006
Zr	0.00000000000000	0.00000000000000	3.50501949847006
Zr	4.95684611216444	3.50501949847006	0.00000000000000
Zr	4.97679368508437	3.53179701781670	7.24383661988189
Zr	0.16684900482869	0.02189427617732	9.85392878524919
O	12.37526021027964	0.14772705136304	6.96774195439332
O	12.39211528041109	0.00000000000000	0.00000000000000
O	17.47897075918448	3.65847750902428	10.32010352400459
O	17.34896139257553	3.50501949847006	3.50501949847006
O	17.32141102248691	-0.02433523163352	7.02145853254495
O	17.34896139257553	0.00000000000000	0.00000000000000
O	12.39211528041109	3.50501949847006	3.50501949847006
Zr	9.91369222432887	0.00000000000000	3.50501949847006
Zr	14.87053833649331	3.50501949847006	0.00000000000000
Zr	14.80032713268158	3.56486471733752	7.34825392635748
Zr	9.85764114655256	-0.00478614715129	9.92828320225161
O	2.36660377174353	7.00695172869300	6.99977149507286
O	2.47842305608222	7.01003899882984	0.00000000000000
O	7.52416508069323	10.57353979121884	10.42324483637798
O	7.43526916824665	10.51505849729990	3.50501949847006
O	7.28374548891288	7.08389480025582	6.97863014058579
O	7.43526916824665	7.01003899882984	0.00000000000000
O	2.34919011746679	10.63652689519788	10.33306040283472
O	2.47842305608222	10.51505849729990	3.50501949847006
Zr	0.00000000000000	7.01003899882984	3.50501949847006
Zr	4.95684611216444	10.51505849729990	0.00000000000000
Zr	4.99552287409275	10.49558847139983	7.26416044672194
Zr	-0.14610956107334	7.05478077061591	9.88120820073683
O	12.39063032417344	6.93645547103367	7.02116350661259
O	12.39211528041109	7.01003899882984	0.00000000000000
O	17.45040048500894	10.34825419016325	10.36421476032316
O	17.34896139257553	10.51505849729990	3.50501949847006
O	17.23857626296707	7.04175811797827	6.88174398420258
O	17.34896139257553	7.01003899882984	0.00000000000000
O	12.28659302083460	10.61472399672734	10.26079465287510
O	12.39211528041109	10.51505849729990	3.50501949847006
Zr	9.91369222432887	7.01003899882984	3.50501949847006
Zr	14.87053833649331	10.51505849729990	0.00000000000000
Zr	14.88869200752059	10.54214703533110	7.27975472937732
Zr	9.92002052216871	7.27138856222264	10.08218077066141
N	12.23558852848468	3.31056319744297	10.87665742502152
O	10.47581462478202	5.31789771850848	13.31123746306272
C	8.11230700488812	6.61164256622217	14.82987069329026
O	6.63262008523365	5.70779076527836	16.18193634804199

Path 1--vii

ATOM		X (Angstroms)	Y (Angstroms)	Z (Angstroms)
1	O	1.398051	-0.002509	3.645508
2	O	1.311525	0.000000	0.000000
3	O	3.925799	1.903735	5.493747
4	O	3.934575	1.854776	1.854776
5	O	3.994052	0.008668	3.689358
6	O	3.934575	0.000000	0.000000
7	O	1.235167	1.771306	5.488584
8	O	1.311525	1.854776	1.854776
9	Zr	0.000000	0.000000	1.854776
10	Zr	2.623050	1.854776	0.000000
11	Zr	2.647493	1.857569	3.867293
12	Zr	0.107360	-0.007546	5.243371
13	O	6.645889	-0.018603	3.633464
14	O	6.557625	0.000000	0.000000
15	O	9.241204	1.949880	5.525709
16	O	9.180675	1.854776	1.854776
17	O	9.238218	-0.003086	3.723590
18	O	9.180675	0.000000	0.000000
19	O	6.557625	1.854776	1.854776
20	Zr	5.246100	0.000000	1.854776
21	Zr	7.869150	1.854776	0.000000

22	Zr	7.829057	1.857742	3.918833
23	Zr	5.354716	0.014397	5.250702
24	O	1.350463	3.708711	3.736299
25	O	1.311525	3.709553	0.000000
26	O	3.947839	5.513621	5.506661
27	O	3.934575	5.564329	1.854776
28	O	3.970208	3.699203	3.654589
29	O	3.934575	3.709553	0.000000
30	O	1.230409	5.648405	5.504617
31	O	1.311525	5.564329	1.854776
32	Zr	0.000000	3.709553	1.854776
33	Zr	2.623050	5.564329	0.000000
34	Zr	2.685475	5.558439	3.912590
35	Zr	-0.063145	3.708824	5.248259
36	O	6.612385	3.732446	3.680419
37	O	6.557625	3.709553	0.000000
38	O	9.223936	5.467524	5.495264
39	O	9.180675	5.564329	1.854776
40	O	9.194166	3.708938	3.636511
41	O	9.180675	3.709553	0.000000
42	O	6.483136	5.611327	5.472542
43	O	6.557625	5.564329	1.854776
44	Zr	5.246100	3.709553	1.854776
45	Zr	7.869150	5.564329	0.000000
46	Zr	7.890788	5.562848	3.877906
47	Zr	5.288125	3.668661	5.267979
48	N	6.452297	1.822195	5.521003
49	O	4.473227	3.716423	7.832024
50	C	3.296805	3.705627	7.931474
51	O	2.140597	3.694843	8.096272

Path 1--viii

ATOM		X (Angstroms)	Y (Angstroms)	Z (Angstroms)
1	O	1.395995	0.002852	3.642681
2	O	1.311525	0.000000	0.000000
3	O	3.941567	1.924818	5.483934
4	O	3.934575	1.854776	1.854776
5	O	3.990534	0.005998	3.703089
6	O	3.934575	0.000000	0.000000
7	O	1.245445	1.764286	5.496132
8	O	1.311525	1.854776	1.854776
9	Zr	0.000000	0.000000	1.854776
10	Zr	2.623050	1.854776	0.000000
11	Zr	2.640552	1.858476	3.867023
12	Zr	0.114571	-0.002699	5.246857
13	O	6.643588	-0.012592	3.637940
14	O	6.557625	0.000000	0.000000
15	O	9.247751	1.950356	5.524809
16	O	9.180675	1.854776	1.854776
17	O	9.236771	-0.010583	3.731718
18	O	9.180675	0.000000	0.000000
19	O	6.557625	1.854776	1.854776
20	Zr	5.246100	0.000000	1.854776
21	Zr	7.869150	1.854776	0.000000
22	Zr	7.822715	1.855946	3.928792
23	Zr	5.359085	0.005069	5.256607
24	O	1.341334	3.705672	3.737590
25	O	1.311525	3.709553	0.000000
26	O	3.972418	5.499426	5.508213
27	O	3.934575	5.564329	1.854776
28	O	3.951660	3.701167	3.642177
29	O	3.934575	3.709553	0.000000
30	O	1.239986	5.654363	5.501938
31	O	1.311525	5.564329	1.854776
32	Zr	0.000000	3.709553	1.854776
33	Zr	2.623050	5.564329	0.000000
34	Zr	2.682901	5.561033	3.919380
35	Zr	-0.072242	3.710192	5.245873

36	O	6.591446	3.728760	3.681169
37	O	6.557625	3.709553	0.000000
38	O	9.230079	5.455991	5.508515
39	O	9.180675	5.564329	1.854776
40	O	9.180493	3.711884	3.636782
41	O	9.180675	3.709553	0.000000
42	O	6.496316	5.618433	5.477137
43	O	6.557625	5.564329	1.854776
44	Zr	5.246100	3.709553	1.854776
45	Zr	7.869150	5.564329	0.000000
46	Zr	7.893732	5.565173	3.886200
47	Zr	5.259514	3.667275	5.252826
48	N	6.458864	1.824515	5.537802

Path 1--ix

ATOM		X (Angstroms)	Y (Angstroms)	Z (Angstroms)
1	O	1.395862	-0.004115	3.639153
2	O	1.311525	0.000000	0.000000
3	O	3.939770	1.937281	5.491200
4	O	3.934575	1.854776	1.854776
5	O	3.987378	0.003480	3.713843
6	O	3.934575	0.000000	0.000000
7	O	1.251538	1.760186	5.492202
8	O	1.311525	1.854776	1.854776
9	Zr	0.000000	0.000000	1.854776
10	Zr	2.623050	1.854776	0.000000
11	Zr	2.646791	1.855498	3.871871
12	Zr	0.114185	0.001295	5.244003
13	O	6.646481	-0.013443	3.631276
14	O	6.557625	0.000000	0.000000
15	O	9.260290	1.952665	5.527357
16	O	9.180675	1.854776	1.854776
17	O	9.234879	-0.005869	3.728729
18	O	9.180675	0.000000	0.000000
19	O	6.557625	1.854776	1.854776
20	Zr	5.246100	0.000000	1.854776
21	Zr	7.869150	1.854776	0.000000
22	Zr	7.824725	1.857875	3.928351
23	Zr	5.372709	0.027321	5.265439
24	O	1.337126	3.712234	3.740035
25	O	1.311525	3.709553	0.000000
26	O	3.973881	5.465931	5.514900
27	O	3.934575	5.564329	1.854776
28	O	3.944631	3.698582	3.636260
29	O	3.934575	3.709553	0.000000
30	O	1.247484	5.661440	5.510531
31	O	1.311525	5.564329	1.854776
32	Zr	0.000000	3.709553	1.854776
33	Zr	2.623050	5.564329	0.000000
34	Zr	2.679741	5.556645	3.924975
35	Zr	-0.074040	3.704557	5.247798
36	O	6.588075	3.734464	3.687052
37	O	6.557625	3.709553	0.000000
38	O	9.237880	5.461265	5.501146
39	O	9.180675	5.564329	1.854776
40	O	9.177752	3.710130	3.637287
41	O	9.180675	3.709553	0.000000
42	O	6.501679	5.629356	5.484064
43	O	6.557625	5.564329	1.854776
44	Zr	5.246100	3.709553	1.854776
45	Zr	7.869150	5.564329	0.000000
46	Zr	7.885660	5.565889	3.888142
47	Zr	5.246182	3.663038	5.250964
48	N	6.468296	1.815898	5.528126
49	O	6.132511	1.798062	10.180726
50	N	6.500867	2.041359	9.105319

Path 1--x

ATOM		X (Angstroms)	Y (Angstroms)	Z (Angstroms)
1	O	1.266823	0.020704	3.586840
2	O	1.311525	0.000000	0.000000
3	O	4.243133	1.861566	5.285628
4	O	3.934575	1.854776	1.854776
5	O	3.809449	-0.096819	3.694301
6	O	3.934575	0.000000	0.000000
7	O	1.397448	1.665901	5.518893
8	O	1.311525	1.854776	1.854776
9	Zr	0.000000	0.000000	1.854776
10	Zr	2.623050	1.854776	0.000000
11	Zr	2.671501	1.815849	3.848503
12	Zr	0.089707	0.036685	5.231307
13	O	6.515189	0.141423	3.782214
14	O	6.557625	0.000000	0.000000
15	O	9.336185	1.919444	5.378591
16	O	9.180675	1.854776	1.854776
17	O	9.133165	-0.017071	3.687878
18	O	9.180675	0.000000	0.000000
19	O	6.557625	1.854776	1.854776
20	Zr	5.246100	0.000000	1.854776
21	Zr	7.869150	1.854776	0.000000
22	Zr	7.875353	1.856219	3.717481
23	Zr	5.167533	-0.223160	5.393674
24	O	1.198969	3.672225	3.708240
25	O	1.311525	3.709553	0.000000
26	O	3.961139	5.506739	5.628795
27	O	3.934575	5.564329	1.854776
28	O	3.786694	3.781362	3.655977
29	O	3.934575	3.709553	0.000000
30	O	1.246500	5.655151	5.420832
31	O	1.311525	5.564329	1.854776
32	Zr	0.000000	3.709553	1.854776
33	Zr	2.623050	5.564329	0.000000
34	Zr	2.544816	5.569619	3.745532
35	Zr	-0.140855	3.767857	5.233246
36	O	6.450939	3.602194	3.655382
37	O	6.557625	3.709553	0.000000
38	O	9.226963	5.493770	5.467493
39	O	9.180675	5.564329	1.854776
40	O	9.056695	3.753492	3.615977
41	O	9.180675	3.709553	0.000000
42	O	6.463837	5.518361	5.321062
43	O	6.557625	5.564329	1.854776
44	Zr	5.246100	3.709553	1.854776
45	Zr	7.869150	5.564329	0.000000
46	Zr	7.874751	5.595248	3.804655
47	Zr	5.142817	3.818118	5.259833
48	N	6.826382	2.362423	5.908029
49	O	6.183358	0.522656	7.037255
50	N	6.370004	1.953103	6.967193

Path 1—TS3

ATOM	X (au)	Y (au)	Z (au)
O	2.39096672187985	0.04407015745206	6.77886058165073
O	2.47842305608222	0.00000000000000	0.00000000000000
O	8.00877252554580	3.52796136700148	9.99143130333760
O	7.43526916824665	3.50501949847006	3.50501949847006
O	7.19084327015392	-0.17594887137948	6.97440335614695
O	7.43526916824665	0.00000000000000	0.00000000000000
O	2.61886686485404	3.15780713510651	10.42876863623244
O	2.47842305608222	3.50501949847006	3.50501949847006
Zr	0.00000000000000	0.00000000000000	3.50501949847006
Zr	4.95684611216444	3.50501949847006	0.00000000000000
Zr	5.04799086861962	3.43079493966750	7.26767575683363
Zr	0.16709267745360	0.06785934516287	9.88925873449575
O	12.31794969240198	0.27832594638257	7.15549129614937
O	12.39211528041109	0.00000000000000	0.00000000000000

O	17.61852876072493	3.63262648271247	10.16206105676840
O	17.34896139257553	3.50501949847006	3.50501949847006
O	17.26129943916323	-0.03220869238536	6.96776539266645
O	17.34896139257553	0.00000000000000	0.00000000000000
O	12.39211528041109	3.50501949847006	3.50501949847006
Zr	9.91369222432887	0.00000000000000	3.50501949847006
Zr	14.87053833649331	3.50501949847006	0.00000000000000
Zr	14.88913253858512	3.50670822198604	7.00674542918166
Zr	9.76916713247093	-0.41111949562436	10.21913618658047
O	2.26096100433205	6.94147615587379	7.00700121684076
O	2.47842305608222	7.01003899882984	0.00000000000000
O	7.46718968117740	10.40011866285758	10.63061046883616
O	7.43526916824665	10.51505849729990	3.50501949847006
O	7.14872972001386	7.14852257957440	6.89791998314074
O	7.43526916824665	7.01003899882984	0.00000000000000
O	2.34616944127859	10.69473099275045	10.24419648534116
O	2.47842305608222	10.51505849729990	3.50501949847006
Zr	0.00000000000000	7.01003899882984	3.50501949847006
Zr	4.95684611216444	10.51505849729990	0.00000000000000
Zr	4.80707278598476	10.53395670152317	7.07983690064077
Zr	-0.27135543645751	7.11363507190548	9.88701365104213
O	12.18656971310375	6.78427926926894	6.92257561972848
O	12.39211528041109	7.01003899882984	0.00000000000000
O	17.42741652660754	10.37748929833545	10.33295872990001
O	17.34896139257553	10.51505849729990	3.50501949847006
O	17.11329176725008	7.09399914127972	6.82598581397565
O	17.34896139257553	7.01003899882984	0.00000000000000
O	12.20299299971290	10.42092209462925	10.04179802256896
O	12.39211528041109	10.51505849729990	3.50501949847006
Zr	9.91369222432887	7.01003899882984	3.50501949847006
Zr	14.87053833649331	10.51505849729990	0.00000000000000
Zr	14.87934754804827	10.57941981011704	7.19571666785013
Zr	9.69116475981093	7.22759975664470	9.93152943992954
N	12.87375877487429	4.56435258114861	11.37758005658999
O	11.62671516163485	0.92438536092756	13.32596078606490
N	12.09379524738974	3.81944398728820	13.36050909545328

Path 1--xi

ATOM		X (Angstroms)	Y (Angstroms)	Z (Angstroms)
1	O	1.239268	0.058891	3.608243
2	O	1.311525	0.000000	0.000000
3	O	4.151612	1.917811	5.304844
4	O	3.934575	1.854776	1.854776
5	O	3.743874	-0.052916	3.651073
6	O	3.934575	0.000000	0.000000
7	O	1.169593	1.753505	5.514537
8	O	1.311525	1.854776	1.854776
9	Zr	0.000000	0.000000	1.854776
10	Zr	2.623050	1.854776	0.000000
11	Zr	2.659860	1.809551	3.801293
12	Zr	0.042350	0.030656	5.263741
13	O	6.554368	0.188737	3.831193
14	O	6.557625	0.000000	0.000000
15	O	9.031718	1.984303	5.410710
16	O	9.180675	1.854776	1.854776
17	O	9.155902	-0.027294	3.683757
18	O	9.180675	0.000000	0.000000
19	O	6.557625	1.854776	1.854776
20	Zr	5.246100	0.000000	1.854776
21	Zr	7.869150	1.854776	0.000000
22	Zr	7.907799	1.833037	3.567002
23	Zr	5.177930	-0.183643	5.547504
24	O	1.155937	3.684279	3.718770
25	O	1.311525	3.709553	0.000000
26	O	3.828378	5.453718	5.591825
27	O	3.934575	5.564329	1.854776
28	O	3.745382	3.796726	3.597047
29	O	3.934575	3.709553	0.000000

30	O	1.156172	5.727875	5.428731
31	O	1.311525	5.564329	1.854776
32	Zr	0.000000	3.709553	1.854776
33	Zr	2.623050	5.564329	0.000000
34	Zr	2.529321	5.631360	3.771948
35	Zr	-0.224517	3.677679	5.217286
36	O	6.404874	3.394430	3.794234
37	O	6.557625	3.709553	0.000000
38	O	9.138162	5.446615	5.492030
39	O	9.180675	5.564329	1.854776
40	O	9.042810	3.755437	3.558908
41	O	9.180675	3.709553	0.000000
42	O	6.383713	5.444289	5.265602
43	O	6.557625	5.564329	1.854776
44	Zr	5.246100	3.709553	1.854776
45	Zr	7.869150	5.564329	0.000000
46	Zr	7.861228	5.627258	3.858743
47	Zr	4.983083	3.831911	5.255501
48	N	6.335705	3.300662	7.483768
49	O	5.802954	0.399727	7.136927
50	N	7.055991	3.172027	8.309562

Path 1--xii

ATOM		X (Angstroms)	Y (Angstroms)	Z (Angstroms)
1	O	1.240120	0.057280	3.607579
2	O	1.311525	0.000000	0.000000
3	O	4.140497	1.927273	5.318788
4	O	3.934575	1.854776	1.854776
5	O	3.748025	-0.042419	3.652932
6	O	3.934575	0.000000	0.000000
7	O	1.174352	1.751910	5.509008
8	O	1.311525	1.854776	1.854776
9	Zr	0.000000	0.000000	1.854776
10	Zr	2.623050	1.854776	0.000000
11	Zr	2.647389	1.804699	3.800989
12	Zr	0.038654	0.026948	5.265867
13	O	6.558187	0.175025	3.832462
14	O	6.557625	0.000000	0.000000
15	O	9.041151	1.980230	5.401485
16	O	9.180675	1.854776	1.854776
17	O	9.154695	-0.028605	3.681358
18	O	9.180675	0.000000	0.000000
19	O	6.557625	1.854776	1.854776
20	Zr	5.246100	0.000000	1.854776
21	Zr	7.869150	1.854776	0.000000
22	Zr	7.913070	1.831496	3.566049
23	Zr	5.201785	-0.170235	5.549369
24	O	1.152504	3.690022	3.722148
25	O	1.311525	3.709553	0.000000
26	O	3.833873	5.458840	5.585775
27	O	3.934575	5.564329	1.854776
28	O	3.745181	3.797193	3.595455
29	O	3.934575	3.709553	0.000000
30	O	1.168761	5.740203	5.435649
31	O	1.311525	5.564329	1.854776
32	Zr	0.000000	3.709553	1.854776
33	Zr	2.623050	5.564329	0.000000
34	Zr	2.530074	5.644878	3.780933
35	Zr	-0.226413	3.674545	5.219284
36	O	6.402554	3.405724	3.785446
37	O	6.557625	3.709553	0.000000
38	O	9.153588	5.453106	5.485241
39	O	9.180675	5.564329	1.854776
40	O	9.037464	3.753342	3.560143
41	O	9.180675	3.709553	0.000000
42	O	6.395294	5.427887	5.303548
43	O	6.557625	5.564329	1.854776
44	Zr	5.246100	3.709553	1.854776

45	Zr	7.869150	5.564329	0.000000
46	Zr	7.849936	5.622856	3.869874
47	Zr	4.969375	3.827306	5.239821
48	O	5.883278	0.489030	7.080887

Path 2--v

ATOM		X (Angstroms)	Y (Angstroms)	Z (Angstroms)
1	O	1.272311	0.042253	3.668802
2	O	1.311525	0.000000	0.000000
3	O	4.082296	1.846626	5.109599
4	O	3.934575	1.854776	1.854776
5	O	3.878900	-0.109490	3.676607
6	O	3.934575	0.000000	0.000000
7	O	1.226974	1.874724	5.512217
8	O	1.311525	1.854776	1.854776
9	Zr	0.000000	0.000000	1.854776
10	Zr	2.623050	1.854776	0.000000
11	Zr	2.551172	1.856634	3.775245
12	Zr	-0.040163	0.003622	5.217423
13	O	6.553524	0.122574	3.779093
14	O	6.557625	0.000000	0.000000
15	O	9.192880	1.836806	5.352960
16	O	9.180675	1.854776	1.854776
17	O	9.144358	-0.039572	3.629445
18	O	9.180675	0.000000	0.000000
19	O	6.557625	1.854776	1.854776
20	Zr	5.246100	0.000000	1.854776
21	Zr	7.869150	1.854776	0.000000
22	Zr	7.959490	1.853577	3.618758
23	Zr	5.163078	-0.039152	5.369837
24	O	1.284338	3.670103	3.647804
25	O	1.311525	3.709553	0.000000
26	O	3.992846	5.609519	5.623152
27	O	3.934575	5.564329	1.854776
28	O	3.882641	3.829446	3.703942
29	O	3.934575	3.709553	0.000000
30	O	1.223393	5.544058	5.409390
31	O	1.311525	5.564329	1.854776
32	Zr	0.000000	3.709553	1.854776
33	Zr	2.623050	5.564329	0.000000
34	Zr	2.665929	5.568065	3.840550
35	Zr	-0.000891	3.715294	5.218548
36	O	6.558991	3.590221	3.760925
37	O	6.557625	3.709553	0.000000
38	O	9.228617	5.579757	5.488022
39	O	9.180675	5.564329	1.854776
40	O	9.154814	3.746805	3.647802
41	O	9.180675	3.709553	0.000000
42	O	6.485792	5.558673	5.467877
43	O	6.557625	5.564329	1.854776
44	Zr	5.246100	3.709553	1.854776
45	Zr	7.869150	5.564329	0.000000
46	Zr	7.818753	5.556748	3.913673
47	Zr	5.182453	3.729055	5.379554
48	O	6.537532	1.954691	5.945810
49	N	5.533982	1.872831	6.847394

Path 2--vi

ATOM		X (Angstroms)	Y (Angstroms)	Z (Angstroms)
1	O	1.245173	0.011522	3.720391
2	O	1.311525	0.000000	0.000000
3	O	3.996290	1.797035	5.344827
4	O	3.934575	1.854776	1.854776
5	O	3.834540	-0.068128	3.650772
6	O	3.934575	0.000000	0.000000
7	O	1.238895	1.972951	5.506408
8	O	1.311525	1.854776	1.854776
9	Zr	0.000000	0.000000	1.854776

10	Zr	2.623050	1.854776	0.000000
11	Zr	2.642598	1.844721	3.833853
12	Zr	-0.131607	0.007545	5.232171
13	O	6.513328	0.062998	3.727875
14	O	6.557625	0.000000	0.000000
15	O	9.219645	1.768573	5.394473
16	O	9.180675	1.854776	1.854776
17	O	9.103285	-0.000717	3.582742
18	O	9.180675	0.000000	0.000000
19	O	6.557625	1.854776	1.854776
20	Zr	5.246100	0.000000	1.854776
21	Zr	7.869150	1.854776	0.000000
22	Zr	7.951262	1.878647	3.690399
23	Zr	5.144705	-0.073328	5.295832
24	O	1.326097	3.687212	3.609090
25	O	1.311525	3.709553	0.000000
26	O	3.978909	5.558520	5.527147
27	O	3.934575	5.564329	1.854776
28	O	3.898661	3.748310	3.691473
29	O	3.934575	3.709553	0.000000
30	O	1.236419	5.450248	5.449776
31	O	1.311525	5.564329	1.854776
32	Zr	0.000000	3.709553	1.854776
33	Zr	2.623050	5.564329	0.000000
34	Zr	2.595352	5.543572	3.811339
35	Zr	0.106061	3.686633	5.241542
36	O	6.577198	3.675784	3.695160
37	O	6.557625	3.709553	0.000000
38	O	9.210025	5.700245	5.454780
39	O	9.180675	5.564329	1.854776
40	O	9.180003	3.725553	3.696742
41	O	9.180675	3.709553	0.000000
42	O	6.476241	5.539320	5.443634
43	O	6.557625	5.564329	1.854776
44	Zr	5.246100	3.709553	1.854776
45	Zr	7.869150	5.564329	0.000000
46	Zr	7.845321	5.575813	3.840678
47	Zr	5.243812	3.811265	5.305121
48	O	6.504266	1.805259	5.548203
49	N	6.219103	2.371140	6.864569
50	C	5.542283	1.440756	7.558367
51	O	5.090961	1.385117	8.678837

Path 2--vii

ATOM		X (Angstroms)	Y (Angstroms)	Z (Angstroms)
1	O	1.228612	0.007772	3.722761
2	O	1.311525	0.000000	0.000000
3	O	4.004384	1.744169	5.384755
4	O	3.934575	1.854776	1.854776
5	O	3.814468	-0.038267	3.607248
6	O	3.934575	0.000000	0.000000
7	O	1.246338	1.984884	5.494756
8	O	1.311525	1.854776	1.854776
9	Zr	0.000000	0.000000	1.854776
10	Zr	2.623050	1.854776	0.000000
11	Zr	2.643433	1.853861	3.841236
12	Zr	-0.156294	-0.009190	5.226378
13	O	6.494532	0.058138	3.709858
14	O	6.557625	0.000000	0.000000
15	O	9.213477	1.765582	5.398185
16	O	9.180675	1.854776	1.854776
17	O	9.087216	-0.005183	3.579845
18	O	9.180675	0.000000	0.000000
19	O	6.557625	1.854776	1.854776
20	Zr	5.246100	0.000000	1.854776
21	Zr	7.869150	1.854776	0.000000
22	Zr	7.951631	1.887928	3.676791
23	Zr	5.118645	-0.069590	5.260044

24	O	1.314354	3.693394	3.599762
25	O	1.311525	3.709553	0.000000
26	O	3.946983	5.629234	5.467325
27	O	3.934575	5.564329	1.854776
28	O	3.879774	3.741238	3.735481
29	O	3.934575	3.709553	0.000000
30	O	1.219970	5.445964	5.452941
31	O	1.311525	5.564329	1.854776
32	Zr	0.000000	3.709553	1.854776
33	Zr	2.623050	5.564329	0.000000
34	Zr	2.579902	5.548551	3.796813
35	Zr	0.099366	3.687278	5.238506
36	O	6.568385	3.661180	3.704405
37	O	6.557625	3.709553	0.000000
38	O	9.190201	5.689738	5.454572
39	O	9.180675	5.564329	1.854776
40	O	9.175485	3.720898	3.691587
41	O	9.180675	3.709553	0.000000
42	O	6.454757	5.526998	5.438818
43	O	6.557625	5.564329	1.854776
44	Zr	5.246100	3.709553	1.854776
45	Zr	7.869150	5.564329	0.000000
46	Zr	7.815218	5.551417	3.838289
47	Zr	5.211194	3.813467	5.384353
48	O	6.505033	1.810894	5.553119
49	N	6.364734	2.187727	6.921986
50	C	5.721141	1.243933	7.612109
51	O	5.330850	1.093231	8.743806
52	N	4.591343	4.034006	7.575985
53	O	4.532351	4.257768	8.719555

Path 2—TS2

ATOM	X (au)	Y (au)	Z (au)
O	2.33652274467901	0.01434981863357	7.03681405586334
O	2.47842305608222	0.00000000000000	0.00000000000000
O	7.55195796500464	3.28483540584095	10.20427486632801
O	7.43526916824665	3.50501949847006	3.50501949847006
O	7.23453479263094	-0.06777721577575	6.82665073867916
O	7.43526916824665	0.00000000000000	0.00000000000000
O	2.35960431499368	3.75008211710390	10.37850292853163
O	2.47842305608222	3.50501949847006	3.50501949847006
Zr	0.00000000000000	0.00000000000000	3.50501949847006
Zr	4.95684611216444	3.50501949847006	0.00000000000000
Zr	4.98270870548985	3.50592011548506	7.26173815609068
Zr	-0.28926452779134	-0.01328821018080	9.87738568729763
O	12.27275929774412	0.08943409510862	7.02002507512366
O	12.39211528041109	0.00000000000000	0.00000000000000
O	17.42579763414105	3.33258267175552	10.21544400188293
O	17.34896139257553	3.50501949847006	3.50501949847006
O	17.17710312441142	-0.01063067132369	6.77348878947604
O	17.34896139257553	0.00000000000000	0.00000000000000
O	12.39211528041109	3.50501949847006	3.50501949847006
Zr	9.91369222432887	0.00000000000000	3.50501949847006
Zr	14.87053833649331	3.50501949847006	0.00000000000000
Zr	14.99827452886979	3.56352633014785	7.00858777987270
Zr	9.67031197217233	-0.08913433541231	9.90586240869162
O	2.49650057492612	6.98225098434265	6.80623089107525
O	2.47842305608222	7.01003899882984	0.00000000000000
O	7.46463092208430	10.65763917813824	10.33227627042778
O	7.43526916824665	10.51505849729990	3.50501949847006
O	7.35166880130508	7.06338173057244	7.06675769416939
O	7.43526916824665	7.01003899882984	0.00000000000000
O	2.31232861700552	10.29146098481231	10.30747193885017
O	2.47842305608222	10.51505849729990	3.50501949847006
Zr	0.00000000000000	7.01003899882984	3.50501949847006
Zr	4.95684611216444	10.51505849729990	0.00000000000000
Zr	4.87956643880478	10.48679957062127	7.18573702418403
Zr	0.18849150145602	6.97346813824659	9.89846575040756

O	12.41183662287824	6.93076977078318	6.99423292322928
O	12.39211528041109	7.01003899882984	0.00000000000000
O	17.37544099848829	10.74961953180165	10.31155045537724
O	17.34896139257553	10.51505849729990	3.50501949847006
O	17.34134495914313	7.03159648423771	6.98453698036688
O	17.34896139257553	7.01003899882984	0.00000000000000
O	12.20168923608530	10.43787213861629	10.28558231139911
O	12.39211528041109	10.51505849729990	3.50501949847006
Zr	9.91369222432887	7.01003899882984	3.50501949847006
Zr	14.87053833649331	10.51505849729990	0.00000000000000
Zr	14.78696128688239	10.49077137620379	7.25598029097892
Zr	9.88526942438013	7.16522350663556	10.15067544363723
O	12.25855361986046	3.42756254423343	10.48070266785570
N	11.90508457927720	4.24466616127378	13.55797586247832
C	10.80865551514034	2.73600675624560	15.04447830239027
O	10.03957861482420	2.36581097116286	17.14577464755782
N	8.71545697145127	7.61265473225855	14.36362407313252
O	8.63116847207964	8.12525163829296	16.50286701462012

Path 2--viii

ATOM		X (Angstroms)	Y (Angstroms)	Z (Angstroms)
1	O	1.282057	0.000497	3.733549
2	O	1.311525	0.000000	0.000000
3	O	3.964721	1.718843	5.471550
4	O	3.934575	1.854776	1.854776
5	O	3.877107	-0.001729	3.605875
6	O	3.934575	0.000000	0.000000
7	O	1.243636	1.974260	5.480903
8	O	1.311525	1.854776	1.854776
9	Zr	0.000000	0.000000	1.854776
10	Zr	2.623050	1.854776	0.000000
11	Zr	2.624838	1.854910	3.864659
12	Zr	-0.127458	0.003076	5.241559
13	O	6.527921	-0.001967	3.715750
14	O	6.557625	0.000000	0.000000
15	O	9.220412	1.741624	5.483314
16	O	9.180675	1.854776	1.854776
17	O	9.121972	0.002874	3.613598
18	O	9.180675	0.000000	0.000000
19	O	6.557625	1.854776	1.854776
20	Zr	5.246100	0.000000	1.854776
21	Zr	7.869150	1.854776	0.000000
22	Zr	7.846583	1.863073	3.858620
23	Zr	5.134550	0.004820	5.245408
24	O	1.363811	3.707742	3.612584
25	O	1.311525	3.709553	0.000000
26	O	3.980713	5.685350	5.467316
27	O	3.934575	5.564329	1.854776
28	O	3.951268	3.707872	3.742493
29	O	3.934575	3.709553	0.000000
30	O	1.251888	5.444351	5.475895
31	O	1.311525	5.564329	1.854776
32	Zr	0.000000	3.709553	1.854776
33	Zr	2.623050	5.564329	0.000000
34	Zr	2.624623	5.560636	3.858926
35	Zr	0.106095	3.710447	5.242525
36	O	6.618144	3.715375	3.623756
37	O	6.557625	3.709553	0.000000
38	O	9.227514	5.678914	5.480595
39	O	9.180675	5.564329	1.854776
40	O	9.206693	3.710262	3.726508
41	O	9.180675	3.709553	0.000000
42	O	6.493112	5.474334	5.460408
43	O	6.557625	5.564329	1.854776
44	Zr	5.246100	3.709553	1.854776
45	Zr	7.869150	5.564329	0.000000
46	Zr	7.855427	5.558056	3.853211
47	Zr	5.339272	3.726712	5.293635

48	O	6.479454	1.947259	5.444751
49	N	5.309477	2.682300	8.622762
50	C	5.159726	3.705675	9.643997
51	O	5.131233	3.767652	10.831676
52	N	5.208597	3.597144	7.736126
53	O	5.047356	4.732316	8.658760

Path 2—TS3

ATOM	X (au)	Y (au)	Z (au)
O	2.42313246456984	0.00202281953598	7.05673890104717
O	2.47842305608222	0.00000000000000	0.00000000000000
O	7.49706273443323	3.25457150997932	10.34142905875870
O	7.43526916824665	3.50501949847006	3.50501949847006
O	7.32348414086316	0.00087118831006	6.81263943424527
O	7.43526916824665	0.00000000000000	0.00000000000000
O	2.35139048244139	3.73236750693422	10.35785018352117
O	2.47842305608222	3.50501949847006	3.50501949847006
Zr	0.00000000000000	0.00000000000000	3.50501949847006
Zr	4.95684611216444	3.50501949847006	0.00000000000000
Zr	4.95513671693437	3.50548071636648	7.28807620261942
Zr	-0.24002146050088	0.00632305951902	9.90093856664623
O	12.34079828972106	-0.00029412642272	7.02094560796230
O	12.39211528041109	0.00000000000000	0.00000000000000
O	17.42427788993517	3.28895308316251	10.36115430540003
O	17.34896139257553	3.50501949847006	3.50501949847006
O	17.23735533737387	0.00491615841897	6.82723260236058
O	17.34896139257553	0.00000000000000	0.00000000000000
O	12.39211528041109	3.50501949847006	3.50501949847006
Zr	9.91369222432887	0.00000000000000	3.50501949847006
Zr	14.87053833649331	3.50501949847006	0.00000000000000
Zr	14.83747417453979	3.52007338559410	7.27538409898584
Zr	9.70035067126137	0.00963003488434	9.89650052972387
O	2.57742655988163	7.00651604855543	6.82775571823623
O	2.47842305608222	7.01003899882984	0.00000000000000
O	7.52467141769061	10.74745452927203	10.32194194595502
O	7.43526916824665	10.51505849729990	3.50501949847006
O	7.46565949245523	7.00752001658194	7.06864855413006
O	7.43526916824665	7.01003899882984	0.00000000000000
O	2.36452272218674	10.28707218659387	10.34779542135188
O	2.47842305608222	10.51505849729990	3.50501949847006
Zr	0.00000000000000	7.01003899882984	3.50501949847006
Zr	4.95684611216444	10.51505849729990	0.00000000000000
Zr	4.95378367491860	10.51035867283800	7.27780054091475
Zr	0.20301705706031	7.01249923327197	9.90276837144552
O	12.51115317303834	7.01968561417120	6.84291952883924
O	12.39211528041109	7.01003899882984	0.00000000000000
O	17.43741060778122	10.73458665087321	10.35479339466672
O	17.34896139257553	10.51505849729990	3.50501949847006
O	17.39656003342009	7.01092749703121	7.04366203405674
O	17.34896139257553	7.01003899882984	0.00000000000000
O	12.26727643880616	10.34072785509304	10.30972699060910
O	12.39211528041109	10.51505849729990	3.50501949847006
Zr	9.91369222432887	7.01003899882984	3.50501949847006
Zr	14.87053833649331	10.51505849729990	0.00000000000000
Zr	14.85318859275412	10.50519400976441	7.26537184431601
Zr	10.09358445902297	7.04868377525376	9.97186962194102
O	12.24225474337074	3.68962128971560	10.29530041885923
N	9.99395457526381	5.23078607035131	16.40509364126986
C	9.73699621193136	7.39756719697348	18.44822336404363
O	9.69623947570041	7.34837881268790	20.69467674249286
N	9.83299493591873	6.78570850882152	14.70475950435619
O	9.57214159570909	9.27508426445639	16.80052382936064

Path 2--ix

ATOM		X (Angstroms)	Y (Angstroms)	Z (Angstroms)
1	O	1.289719	0.000050	3.744909
2	O	1.311525	0.000000	0.000000
3	O	3.973576	1.724038	5.487789

4	O	3.934575	1.854776	1.854776
5	O	3.874028	0.003381	3.611420
6	O	3.934575	0.000000	0.000000
7	O	1.251125	1.980130	5.491583
8	O	1.311525	1.854776	1.854776
9	Zr	0.000000	0.000000	1.854776
10	Zr	2.623050	1.854776	0.000000
11	Zr	2.621169	1.855475	3.844377
12	Zr	-0.125835	0.000438	5.242155
13	O	6.536189	0.000837	3.730926
14	O	6.557625	0.000000	0.000000
15	O	9.224868	1.731569	5.488306
16	O	9.180675	1.854776	1.854776
17	O	9.120695	-0.001255	3.616124
18	O	9.180675	0.000000	0.000000
19	O	6.557625	1.854776	1.854776
20	Zr	5.246100	0.000000	1.854776
21	Zr	7.869150	1.854776	0.000000
22	Zr	7.860811	1.857578	3.839813
23	Zr	5.127236	0.004719	5.235414
24	O	1.371109	3.707588	3.617909
25	O	1.311525	3.709553	0.000000
26	O	3.978554	5.694117	5.482959
27	O	3.934575	5.564329	1.854776
28	O	3.953363	3.709710	3.745328
29	O	3.934575	3.709553	0.000000
30	O	1.253567	5.437752	5.487952
31	O	1.311525	5.564329	1.854776
32	Zr	0.000000	3.709553	1.854776
33	Zr	2.623050	5.564329	0.000000
34	Zr	2.620380	5.562090	3.841466
35	Zr	0.114074	3.709010	5.243203
36	O	6.619756	3.710134	3.621848
37	O	6.557625	3.709553	0.000000
38	O	9.227018	5.686136	5.489564
39	O	9.180675	5.564329	1.854776
40	O	9.200371	3.709579	3.739707
41	O	9.180675	3.709553	0.000000
42	O	6.497372	5.452587	5.477340
43	O	6.557625	5.564329	1.854776
44	Zr	5.246100	3.709553	1.854776
45	Zr	7.869150	5.564329	0.000000
46	Zr	7.866471	5.559948	3.837464
47	Zr	5.356696	3.714571	5.258099
48	O	6.491865	1.963769	5.474459
49	N	5.024868	3.743977	8.993304
50	C	5.139821	5.958793	11.434779
51	O	5.042540	5.180011	12.308133
52	N	5.120795	3.717033	7.889749
53	O	5.237672	6.739331	10.562281

N_2O formation on ZrO_2 (110) surface--vi

ATOM		X (Angstroms)	Y (Angstroms)	Z (Angstroms)
1	O	1.238832	0.020284	3.714816
2	O	1.311525	0.000000	0.000000
3	O	4.110463	1.795960	5.254025
4	O	3.934575	1.854776	1.854776
5	O	3.831836	-0.092365	3.640210
6	O	3.934575	0.000000	0.000000
7	O	1.307418	1.994158	5.505680
8	O	1.311525	1.854776	1.854776
9	Zr	0.000000	0.000000	1.854776
10	Zr	2.623050	1.854776	0.000000
11	Zr	2.641764	1.850248	3.819343
12	Zr	-0.136978	-0.017838	5.233116
13	O	6.509703	0.080533	3.775992
14	O	6.557625	0.000000	0.000000
15	O	9.271777	1.725247	5.391055

16	O	9.180675	1.854776	1.854776
17	O	9.089946	-0.019816	3.579884
18	O	9.180675	0.000000	0.000000
19	O	6.557625	1.854776	1.854776
20	Zr	5.246100	0.000000	1.854776
21	Zr	7.869150	1.854776	0.000000
22	Zr	7.921799	1.863783	3.699583
23	Zr	5.123567	-0.126121	5.317186
24	O	1.307619	3.676082	3.588017
25	O	1.311525	3.709553	0.000000
26	O	3.958733	5.637362	5.593978
27	O	3.934575	5.564329	1.854776
28	O	3.864238	3.798021	3.715134
29	O	3.934575	3.709553	0.000000
30	O	1.247889	5.428669	5.428867
31	O	1.311525	5.564329	1.854776
32	Zr	0.000000	3.709553	1.854776
33	Zr	2.623050	5.564329	0.000000
34	Zr	2.572179	5.561965	3.769232
35	Zr	0.116238	3.675138	5.236595
36	O	6.534240	3.680131	3.664288
37	O	6.557625	3.709553	0.000000
38	O	9.216045	5.679426	5.456510
39	O	9.180675	5.564329	1.854776
40	O	9.161948	3.716148	3.706766
41	O	9.180675	3.709553	0.000000
42	O	6.468313	5.527006	5.428533
43	O	6.557625	5.564329	1.854776
44	Zr	5.246100	3.709553	1.854776
45	Zr	7.869150	5.564329	0.000000
46	Zr	7.851714	5.550382	3.833936
47	Zr	5.210113	3.828681	5.319019
48	O	6.693885	2.234741	5.600897
49	N	6.273437	1.478231	6.732550
50	N	5.542241	2.353854	7.410000
51	O	4.970816	1.963610	8.438387

N_2O formation on ZrO_2 (110) surface—TS2

ATOM	X (au)	Y (au)	Z (au)
O	2.37375903830211	0.02807487869241	7.02627432760942
O	2.47842305608222	0.00000000000000	0.00000000000000
O	7.70270294489409	3.36963211870801	10.06154281275788
O	7.43526916824665	3.50501949847006	3.50501949847006
O	7.25915387624625	-0.13215248988950	6.85981444384604
O	7.43526916824665	0.00000000000000	0.00000000000000
O	2.45822843359679	3.76191170060115	10.38954963817954
O	2.47842305608222	3.50501949847006	3.50501949847006
Zr	0.00000000000000	0.00000000000000	3.50501949847006
Zr	4.95684611216444	3.50501949847006	0.00000000000000
Zr	4.99272534243457	3.49417297506947	7.24849162381956
Zr	-0.24986646496002	-0.02189053451959	9.89646060170057
O	12.31240622049856	0.10188741105931	7.11172505390136
O	12.39211528041109	0.00000000000000	0.00000000000000
O	17.52642742490528	3.25622770563951	10.23071128255019
O	17.34896139257553	3.50501949847006	3.50501949847006
O	17.19942194079080	-0.03306259537056	6.77799386632241
O	17.34896139257553	0.00000000000000	0.00000000000000
O	12.39211528041109	3.50501949847006	3.50501949847006
Zr	9.91369222432887	0.00000000000000	3.50501949847006
Zr	14.87053833649331	3.50501949847006	0.00000000000000
Zr	14.91948904236524	3.51612740500880	7.12727799690803
Zr	9.70661909312894	-0.17177968012375	10.03264294955916
O	2.51087778891933	6.96340184685822	6.78656461333765
O	2.47842305608222	7.01003899882984	0.00000000000000
O	7.49724271761856	10.66781265479243	10.50946878757765
O	7.43526916824665	10.51505849729990	3.50501949847006
O	7.36578549576497	7.13535099384140	7.03529382221839
O	7.43526916824665	7.01003899882984	0.00000000000000

O	2.37215165993690	10.26399428612733	10.28053083536151
O	2.47842305608222	10.51505849729990	3.50501949847006
Zr	0.00000000000000	7.01003899882984	3.50501949847006
Zr	4.95684611216444	10.51505849729990	0.00000000000000
Zr	4.88499342421040	10.51375098445566	7.16760092383026
Zr	0.22229863191229	6.95536747403711	9.90034116563661
O	12.37487767490988	6.98268496561644	6.90420460185093
O	12.39211528041109	7.01003899882984	0.00000000000000
O	17.42889026543091	10.73541258780149	10.32086850970643
O	17.34896139257553	10.51505849729990	3.50501949847006
O	17.34157679074414	7.02287497676194	7.01905793219015
O	17.34896139257553	7.01003899882984	0.00000000000000
O	12.24304810708980	10.40071633976722	10.28464176447109
O	12.39211528041109	10.51505849729990	3.50501949847006
Zr	9.91369222432887	7.01003899882984	3.50501949847006
Zr	14.87053833649331	10.51505849729990	0.00000000000000
Zr	14.85799194019260	10.50238608650227	7.25749774105730
Zr	9.94531982971025	7.15775689258008	9.99216288057127
O	12.61936963404850	4.05061238324967	10.44973198229798
N	11.62704162883048	2.37216047739900	13.36022010908562
N	10.51399901478902	4.11093784766591	14.40588382949313
O	9.02452725388604	4.26035065945436	16.24920690556691

N_2O formation on ZrO_2 (110) surface--vii

ATOM		X (Angstroms)	Y (Angstroms)	Z (Angstroms)
1	O	1.292585	-0.000414	3.739513
2	O	1.311525	0.000000	0.000000
3	O	3.995985	1.731074	5.478492
4	O	3.934575	1.854776	1.854776
5	O	3.875404	-0.004246	3.618987
6	O	3.934575	0.000000	0.000000
7	O	1.264754	1.979705	5.490440
8	O	1.311525	1.854776	1.854776
9	Zr	0.000000	0.000000	1.854776
10	Zr	2.623050	1.854776	0.000000
11	Zr	2.633945	1.851116	3.841263
12	Zr	-0.118226	0.000332	5.241038
13	O	6.538670	-0.000465	3.746341
14	O	6.557625	0.000000	0.000000
15	O	9.233608	1.724031	5.488604
16	O	9.180675	1.854776	1.854776
17	O	9.122202	0.000766	3.612424
18	O	9.180675	0.000000	0.000000
19	O	6.557625	1.854776	1.854776
20	Zr	5.246100	0.000000	1.854776
21	Zr	7.869150	1.854776	0.000000
22	Zr	7.866397	1.857223	3.841270
23	Zr	5.131368	-0.021996	5.256228
24	O	1.376147	3.706719	3.613133
25	O	1.311525	3.709553	0.000000
26	O	3.987897	5.676615	5.478150
27	O	3.934575	5.564329	1.854776
28	O	3.958116	3.710250	3.733932
29	O	3.934575	3.709553	0.000000
30	O	1.263425	5.437593	5.484252
31	O	1.311525	5.564329	1.854776
32	Zr	0.000000	3.709553	1.854776
33	Zr	2.623050	5.564329	0.000000
34	Zr	2.623685	5.565025	3.831986
35	Zr	0.127640	3.709261	5.240715
36	O	6.620688	3.715579	3.607288
37	O	6.557625	3.709553	0.000000
38	O	9.234925	5.690971	5.486371
39	O	9.180675	5.564329	1.854776
40	O	9.207341	3.709931	3.740471
41	O	9.180675	3.709553	0.000000
42	O	6.511180	5.442111	5.480862
43	O	6.557625	5.564329	1.854776

44	Zr	5.246100	3.709553	1.854776
45	Zr	7.869150	5.564329	0.000000
46	Zr	7.874500	5.566560	3.833830
47	Zr	5.367641	3.723583	5.239465
48	O	6.516046	1.998983	5.483086
49	N	5.396405	0.493832	7.972763
50	N	5.379327	1.613994	8.182612
51	O	5.355331	2.787088	8.369888

CO C-end adsorbed on Zr_T site of ZrO_2 (111) surface

ATOM		X (Angstroms)	Y (Angstroms)	Z (Angstroms)
1	O	-0.001563	4.179113	0.018647
2	O	-0.001563	0.002651	2.971852
3	O	3.615244	2.135485	5.914082
4	O	3.615359	4.179113	0.018647
5	O	3.615359	0.002651	2.971852
6	O	-0.004266	2.092101	5.841297
7	O	5.423820	1.046766	0.018647
8	O	1.806898	3.134997	2.971852
9	O	-1.847622	5.202596	5.913873
10	O	1.806898	1.046766	0.018647
11	O	-1.810024	3.134997	2.971852
12	O	1.844544	5.202422	5.914547
13	O	-0.001563	2.090882	1.495250
14	O	3.615359	4.179113	4.448454
15	O	3.613612	0.007296	7.418469
16	O	3.615359	2.090882	1.495250
17	O	-0.001563	4.179113	4.448454
18	O	-0.001106	0.095941	7.459775
19	O	1.806898	5.223228	1.495250
20	O	1.806898	1.046766	4.448454
21	O	-1.734189	3.093594	7.457442
22	O	-1.810024	5.223228	1.495250
23	O	5.423820	1.046766	4.448454
24	O	1.725833	3.089738	7.458842
25	Zr	-0.001563	0.002651	0.756949
26	Zr	3.615359	2.090882	3.710153
27	Zr	0.002378	4.155389	6.634802
28	Zr	-1.810024	3.134997	0.756949
29	Zr	1.806898	5.223228	3.710153
30	Zr	1.790087	1.054546	6.636804
31	Zr	1.806898	3.134997	0.756949
32	Zr	-1.810024	5.223228	3.710153
33	Zr	5.439947	1.049485	6.635077
34	Zr	3.615359	0.002651	0.756949
35	Zr	-0.001563	2.090882	3.710153
36	Zr	3.623307	4.174295	6.658050
37	C	3.631422	4.165252	9.223621
38	O	3.645653	4.153545	10.360767

CO C-end adsorbed on O_T site of ZrO_2 (111) surface

ATOM		X (Angstroms)	Y (Angstroms)	Z (Angstroms)
1	O	-0.001563	4.179113	0.018647
2	O	-0.001563	0.002651	2.971852
3	O	3.622409	2.088182	5.887272
4	O	3.615359	4.179113	0.018647
5	O	3.615359	0.002651	2.971852
6	O	-0.009665	2.089982	5.889239
7	O	5.423820	1.046766	0.018647
8	O	1.806898	3.134997	2.971852
9	O	-1.809892	5.222582	5.882522
10	O	1.806898	1.046766	0.018647
11	O	-1.810024	3.134997	2.971852
12	O	1.804796	5.230242	5.890407
13	O	-0.001563	2.090882	1.495250
14	O	3.615359	4.179113	4.448454
15	O	3.604414	0.001891	7.441506

16	O	3.615359	2.090882	1.495250
17	O	-0.001563	4.179113	4.448454
18	O	0.006370	0.000519	7.439925
19	O	1.806898	5.223228	1.495250
20	O	1.806898	1.046766	4.448454
21	O	-1.812626	3.147082	7.443105
22	O	-1.810024	5.223228	1.495250
23	O	5.423820	1.046766	4.448454
24	O	1.808641	3.133022	7.422641
25	Zr	-0.001563	0.002651	0.756949
26	Zr	3.615359	2.090882	3.710153
27	Zr	-0.004555	4.178232	6.636731
28	Zr	-1.810024	3.134997	0.756949
29	Zr	1.806898	5.223228	3.710153
30	Zr	1.807703	1.042635	6.635678
31	Zr	1.806898	3.134997	0.756949
32	Zr	-1.810024	5.223228	3.710153
33	Zr	5.424072	1.044168	6.627413
34	Zr	3.615359	0.002651	0.756949
35	Zr	-0.001563	2.090882	3.710153
36	Zr	3.618131	4.177137	6.635883
37	C	1.635959	3.385674	11.246572
38	O	1.797628	3.182400	12.357187

CO C-end adsorbed on O_H site of ZrO_2 (111) surface

ATOM		X (Angstroms)	Y (Angstroms)	Z (Angstroms)
1	O	-0.001563	4.179113	0.018647
2	O	-0.001563	0.002651	2.971852
3	O	3.614474	2.092447	5.912506
4	O	3.615359	4.179113	0.018647
5	O	3.615359	0.002651	2.971852
6	O	-0.002341	2.059081	5.880201
7	O	5.423820	1.046766	0.018647
8	O	1.806898	3.134997	2.971852
9	O	-1.839896	5.240858	5.880200
10	O	1.806898	1.046766	0.018647
11	O	-1.810024	3.134997	2.971852
12	O	1.834662	5.240588	5.880319
13	O	-0.001563	2.090882	1.495250
14	O	3.615359	4.179113	4.448454
15	O	3.613816	-0.037249	7.429780
16	O	3.615359	2.090882	1.495250
17	O	-0.001563	4.179113	4.448454
18	O	-0.002989	0.005076	7.473239
19	O	1.806898	5.223228	1.495250
20	O	1.806898	1.046766	4.448454
21	O	-1.773466	3.159897	7.429760
22	O	-1.810024	5.223228	1.495250
23	O	5.423820	1.046766	4.448454
24	O	1.767737	3.159572	7.429963
25	Zr	-0.001563	0.002651	0.756949
26	Zr	3.615359	2.090882	3.710153
27	Zr	-0.001465	4.175601	6.663334
28	Zr	-1.810024	3.134997	0.756949
29	Zr	1.806898	5.223228	3.710153
30	Zr	1.795596	1.036415	6.626692
31	Zr	1.806898	3.134997	0.756949
32	Zr	-1.810024	5.223228	3.710153
33	Zr	5.435902	1.036357	6.626591
34	Zr	3.615359	0.002651	0.756949
35	Zr	-0.001563	2.090882	3.710153
36	Zr	3.615708	4.189783	6.626901
37	C	3.618190	2.123195	10.326388
38	O	3.618482	2.122564	11.466484

CO O-end adsorbed on Zr_T site of ZrO_2 (111) surface

ATOM		X (Angstroms)	Y (Angstroms)	Z (Angstroms)

1	O	-0.001563	4.179113	0.018647
2	O	-0.001563	0.002651	2.971852
3	O	3.614452	2.112297	5.893248
4	O	3.615359	4.179113	0.018647
5	O	3.615359	0.002651	2.971852
6	O	-0.003349	2.092371	5.868735
7	O	5.423820	1.046766	0.018647
8	O	1.806898	3.134997	2.971852
9	O	-1.828354	5.213990	5.893238
10	O	1.806898	1.046766	0.018647
11	O	-1.810024	3.134997	2.971852
12	O	1.824099	5.213752	5.894194
13	O	-0.001563	2.090882	1.495250
14	O	3.615359	4.179113	4.448454
15	O	3.613274	0.005903	7.422870
16	O	3.615359	2.090882	1.495250
17	O	-0.001563	4.179113	4.448454
18	O	-0.001137	0.037450	7.443027
19	O	1.806898	5.223228	1.495250
20	O	1.806898	1.046766	4.448454
21	O	-1.785851	3.122530	7.440267
22	O	-1.810024	5.223228	1.495250
23	O	5.423820	1.046766	4.448454
24	O	1.778890	3.119502	7.443805
25	Zr	-0.001563	0.002651	0.756949
26	Zr	3.615359	2.090882	3.710153
27	Zr	-0.000630	4.166521	6.636992
28	Zr	-1.810024	3.134997	0.756949
29	Zr	1.806898	5.223228	3.710153
30	Zr	1.797931	1.050295	6.640696
31	Zr	1.806898	3.134997	0.756949
32	Zr	-1.810024	5.223228	3.710153
33	Zr	5.432078	1.048797	6.637090
34	Zr	3.615359	0.002651	0.756949
35	Zr	-0.001563	2.090882	3.710153
36	Zr	3.619978	4.175101	6.620386
37	O	3.669432	4.188891	10.127492
38	C	3.510987	4.215680	11.255531

CO O-end adsorbed on O_T site of ZrO_2 (111) surface

ATOM		X (Angstroms)	Y (Angstroms)	Z (Angstroms)
1	O	-0.001563	4.179113	0.018647
2	O	-0.001563	0.002651	2.971852
3	O	3.622942	2.087620	5.889548
4	O	3.615359	4.179113	0.018647
5	O	3.615359	0.002651	2.971852
6	O	-0.010842	2.088348	5.890141
7	O	5.423820	1.046766	0.018647
8	O	1.806898	3.134997	2.971852
9	O	-1.810548	5.223311	5.877437
10	O	1.806898	1.046766	0.018647
11	O	-1.810024	3.134997	2.971852
12	O	1.805406	5.232566	5.891074
13	O	-0.001563	2.090882	1.495250
14	O	3.615359	4.179113	4.448454
15	O	3.602780	-0.000178	7.442558
16	O	3.615359	2.090882	1.495250
17	O	-0.001563	4.179113	4.448454
18	O	0.007864	-0.000499	7.441880
19	O	1.806898	5.223228	1.495250
20	O	1.806898	1.046766	4.448454
21	O	-1.811863	3.149865	7.443340
22	O	-1.810024	5.223228	1.495250
23	O	5.423820	1.046766	4.448454
24	O	1.806479	3.135432	7.424642
25	Zr	-0.001563	0.002651	0.756949
26	Zr	3.615359	2.090882	3.710153
27	Zr	-0.005838	4.178911	6.635739

28	Zr	-1.810024	3.134997	0.756949
29	Zr	1.806898	5.223228	3.710153
30	Zr	1.807361	1.039635	6.636464
31	Zr	1.806898	3.134997	0.756949
32	Zr	-1.810024	5.223228	3.710153
33	Zr	5.424067	1.044249	6.625693
34	Zr	3.615359	0.002651	0.756949
35	Zr	-0.001563	2.090882	3.710153
36	Zr	3.619932	4.178393	6.636122
37	O	1.743273	3.272407	10.923950
38	C	1.753974	3.231970	12.063125

CO O-end adsorbed on O_H site of ZrO_2 (111) surface

ATOM		X (Angstroms)	Y (Angstroms)	Z (Angstroms)
1	O	-0.001563	4.179113	0.018647
2	O	-0.001563	0.002651	2.971852
3	O	3.614404	2.092256	5.901063
4	O	3.615359	4.179113	0.018647
5	O	3.615359	0.002651	2.971852
6	O	-0.002469	2.074485	5.882995
7	O	5.423820	1.046766	0.018647
8	O	1.806898	3.134997	2.971852
9	O	-1.826337	5.233163	5.882807
10	O	1.806898	1.046766	0.018647
11	O	-1.810024	3.134997	2.971852
12	O	1.821152	5.233016	5.882863
13	O	-0.001563	2.090882	1.495250
14	O	3.615359	4.179113	4.448454
15	O	3.613801	-0.017352	7.433617
16	O	3.615359	2.090882	1.495250
17	O	-0.001563	4.179113	4.448454
18	O	-0.003037	0.004947	7.455837
19	O	1.806898	5.223228	1.495250
20	O	1.806898	1.046766	4.448454
21	O	-1.791112	3.149600	7.433166
22	O	-1.810024	5.223228	1.495250
23	O	5.423820	1.046766	4.448454
24	O	1.785256	3.149404	7.433303
25	Zr	-0.001563	0.002651	0.756949
26	Zr	3.615359	2.090882	3.710153
27	Zr	-0.001326	4.175948	6.650081
28	Zr	-1.810024	3.134997	0.756949
29	Zr	1.806898	5.223228	3.710153
30	Zr	1.800777	1.039769	6.629072
31	Zr	1.806898	3.134997	0.756949
32	Zr	-1.810024	5.223228	3.710153
33	Zr	5.430550	1.039772	6.629019
34	Zr	3.615359	0.002651	0.756949
35	Zr	-0.001563	2.090882	3.710153
36	Zr	3.615701	4.184332	6.629028
37	O	3.618441	2.126700	10.279739
38	C	3.617694	2.122991	11.419341

NO N-end adsorbed on Zr_T site of ZrO_2 (111) surface

ATOM		X (Angstroms)	Y (Angstroms)	Z (Angstroms)
1	O	-0.001563	4.179113	0.018647
2	O	-0.001563	0.002651	2.971852
3	O	3.608603	2.111320	5.899914
4	O	3.615359	4.179113	0.018647
5	O	3.615359	0.002651	2.971852
6	O	-0.003227	2.088865	5.867134
7	O	5.423820	1.046766	0.018647
8	O	1.806898	3.134997	2.971852
9	O	-1.822212	5.219887	5.905757
10	O	1.806898	1.046766	0.018647
11	O	-1.810024	3.134997	2.971852
12	O	1.818779	5.213046	5.899341

13	O	-0.001563	2.090882	1.495250
14	O	3.615359	4.179113	4.448454
15	O	3.617572	0.010864	7.439999
16	O	3.615359	2.090882	1.495250
17	O	-0.001563	4.179113	4.448454
18	O	-0.011618	0.046104	7.451694
19	O	1.806898	5.223228	1.495250
20	O	1.806898	1.046766	4.448454
21	O	-1.780855	3.114575	7.447235
22	O	-1.810024	5.223228	1.495250
23	O	5.423820	1.046766	4.448454
24	O	1.760102	3.111808	7.439862
25	Zr	-0.001563	0.002651	0.756949
26	Zr	3.615359	2.090882	3.710153
27	Zr	0.003118	4.160788	6.630135
28	Zr	-1.810024	3.134997	0.756949
29	Zr	1.806898	5.223228	3.710153
30	Zr	1.802943	1.050314	6.631007
31	Zr	1.806898	3.134997	0.756949
32	Zr	-1.810024	5.223228	3.710153
33	Zr	5.421125	1.039783	6.626792
34	Zr	3.615359	0.002651	0.756949
35	Zr	-0.001563	2.090882	3.710153
36	Zr	3.626031	4.177184	6.668568
37	N	3.654060	4.281330	9.365346
38	O	2.896309	3.979981	10.185726

NO N-end adsorbed on O_T site of ZrO_2 (111) surface

ATOM		X (Angstroms)	Y (Angstroms)	Z (Angstroms)
1	O	-0.001563	4.179113	0.018647
2	O	-0.001563	0.002651	2.971852
3	O	3.624101	2.086727	5.889138
4	O	3.615359	4.179113	0.018647
5	O	3.615359	0.002651	2.971852
6	O	-0.011756	2.087887	5.890266
7	O	5.423820	1.046766	0.018647
8	O	1.806898	3.134997	2.971852
9	O	-1.810190	5.223329	5.878654
10	O	1.806898	1.046766	0.018647
11	O	-1.810024	3.134997	2.971852
12	O	1.805177	5.233792	5.890802
13	O	-0.001563	2.090882	1.495250
14	O	3.615359	4.179113	4.448454
15	O	3.602435	-0.000310	7.444147
16	O	3.615359	2.090882	1.495250
17	O	-0.001563	4.179113	4.448454
18	O	0.008404	-0.001077	7.442961
19	O	1.806898	5.223228	1.495250
20	O	1.806898	1.046766	4.448454
21	O	-1.812195	3.150297	7.444513
22	O	-1.810024	5.223228	1.495250
23	O	5.423820	1.046766	4.448454
24	O	1.807340	3.135434	7.423115
25	Zr	-0.001563	0.002651	0.756949
26	Zr	3.615359	2.090882	3.710153
27	Zr	-0.005621	4.178988	6.635874
28	Zr	-1.810024	3.134997	0.756949
29	Zr	1.806898	5.223228	3.710153
30	Zr	1.807412	1.040121	6.636824
31	Zr	1.806898	3.134997	0.756949
32	Zr	-1.810024	5.223228	3.710153
33	Zr	5.423949	1.044372	6.625322
34	Zr	3.615359	0.002651	0.756949
35	Zr	-0.001563	2.090882	3.710153
36	Zr	3.619761	4.178317	6.636018
37	N	1.676245	3.235236	10.910943
38	O	1.803052	3.272404	12.064501

NO N-end adsorbed on O_H site of ZrO_2 (111) surface

ATOM		X (Angstroms)	Y (Angstroms)	Z (Angstroms)
1	O	-0.001563	4.179113	0.018647
2	O	-0.001563	0.002651	2.971852
3	O	3.614324	2.092683	5.912499
4	O	3.615359	4.179113	0.018647
5	O	3.615359	0.002651	2.971852
6	O	-0.002599	2.061828	5.881179
7	O	5.423820	1.046766	0.018647
8	O	1.806898	3.134997	2.971852
9	O	-1.838273	5.239659	5.880837
10	O	1.806898	1.046766	0.018647
11	O	-1.810024	3.134997	2.971852
12	O	1.832749	5.239499	5.880855
13	O	-0.001563	2.090882	1.495250
14	O	3.615359	4.179113	4.448454
15	O	3.613612	-0.034436	7.429230
16	O	3.615359	2.090882	1.495250
17	O	-0.001563	4.179113	4.448454
18	O	-0.003257	0.005502	7.470115
19	O	1.806898	5.223228	1.495250
20	O	1.806898	1.046766	4.448454
21	O	-1.776621	3.159073	7.431573
22	O	-1.810024	5.223228	1.495250
23	O	5.423820	1.046766	4.448454
24	O	1.769988	3.159045	7.430750
25	Zr	-0.001563	0.002651	0.756949
26	Zr	3.615359	2.090882	3.710153
27	Zr	-0.001059	4.174990	6.662471
28	Zr	-1.810024	3.134997	0.756949
29	Zr	1.806898	5.223228	3.710153
30	Zr	1.796194	1.035365	6.626571
31	Zr	1.806898	3.134997	0.756949
32	Zr	-1.810024	5.223228	3.710153
33	Zr	5.435591	1.035411	6.626436
34	Zr	3.615359	0.002651	0.756949
35	Zr	-0.001563	2.090882	3.710153
36	Zr	3.615920	4.188539	6.626527
37	N	3.598009	2.097440	10.077639
38	O	3.648886	2.198587	11.232416

NO O-end adsorbed on Zr_T site of ZrO_2 (111) surface

ATOM		X (Angstroms)	Y (Angstroms)	Z (Angstroms)
1	O	-0.001563	4.179113	0.018647
2	O	-0.001563	0.002651	2.971852
3	O	3.618916	2.099359	5.892507
4	O	3.615359	4.179113	0.018647
5	O	3.615359	0.002651	2.971852
6	O	-0.012062	2.088119	5.881834
7	O	5.423820	1.046766	0.018647
8	O	1.806898	3.134997	2.971852
9	O	-1.818124	5.218313	5.883692
10	O	1.806898	1.046766	0.018647
11	O	-1.810024	3.134997	2.971852
12	O	1.813702	5.222724	5.894058
13	O	-0.001563	2.090882	1.495250
14	O	3.615359	4.179113	4.448454
15	O	3.607458	0.001909	7.434213
16	O	3.615359	2.090882	1.495250
17	O	-0.001563	4.179113	4.448454
18	O	0.006440	0.011128	7.443436
19	O	1.806898	5.223228	1.495250
20	O	1.806898	1.046766	4.448454
21	O	-1.803422	3.142023	7.446533
22	O	-1.810024	5.223228	1.495250
23	O	5.423820	1.046766	4.448454
24	O	1.787153	3.123627	7.439942

25	Zr	-0.001563	0.002651	0.756949
26	Zr	3.615359	2.090882	3.710153
27	Zr	-0.002191	4.172030	6.638985
28	Zr	-1.810024	3.134997	0.756949
29	Zr	1.806898	5.223228	3.710153
30	Zr	1.802380	1.048655	6.640927
31	Zr	1.806898	3.134997	0.756949
32	Zr	-1.810024	5.223228	3.710153
33	Zr	5.424155	1.045079	6.628166
34	Zr	3.615359	0.002651	0.756949
35	Zr	-0.001563	2.090882	3.710153
36	Zr	3.629145	4.184179	6.627158
37	O	3.353148	4.256841	10.431883
38	N	2.665285	3.750079	11.216997

NO O-end adsorbed on O_T site of ZrO_2 (111) surface

ATOM		X (Angstroms)	Y (Angstroms)	Z (Angstroms)
1	O	-0.001563	4.179113	0.018647
2	O	-0.001563	0.002651	2.971852
3	O	3.623538	2.087009	5.889095
4	O	3.615359	4.179113	0.018647
5	O	3.615359	0.002651	2.971852
6	O	-0.011276	2.087755	5.889759
7	O	5.423820	1.046766	0.018647
8	O	1.806898	3.134997	2.971852
9	O	-1.810597	5.223855	5.876757
10	O	1.806898	1.046766	0.018647
11	O	-1.810024	3.134997	2.971852
12	O	1.805466	5.233491	5.890308
13	O	-0.001563	2.090882	1.495250
14	O	3.615359	4.179113	4.448454
15	O	3.605255	0.000827	7.445292
16	O	3.615359	2.090882	1.495250
17	O	-0.001563	4.179113	4.448454
18	O	0.005549	0.000577	7.444632
19	O	1.806898	5.223228	1.495250
20	O	1.806898	1.046766	4.448454
21	O	-1.811702	3.147522	7.445671
22	O	-1.810024	5.223228	1.495250
23	O	5.423820	1.046766	4.448454
24	O	1.806509	3.136307	7.434935
25	Zr	-0.001563	0.002651	0.756949
26	Zr	3.615359	2.090882	3.710153
27	Zr	-0.004644	4.178034	6.634564
28	Zr	-1.810024	3.134997	0.756949
29	Zr	1.806898	5.223228	3.710153
30	Zr	1.807418	1.041186	6.635414
31	Zr	1.806898	3.134997	0.756949
32	Zr	-1.810024	5.223228	3.710153
33	Zr	5.424096	1.043930	6.628068
34	Zr	3.615359	0.002651	0.756949
35	Zr	-0.001563	2.090882	3.710153
36	Zr	3.618601	4.177430	6.634952
37	O	1.719272	3.220003	10.823389
38	N	1.785249	3.269787	11.980858

NO O-end adsorbed on O_H site of ZrO_2 (111) surface

ATOM		X (Angstroms)	Y (Angstroms)	Z (Angstroms)
1	O	-0.001563	4.179113	0.018647
2	O	-0.001563	0.002651	2.971852
3	O	3.614324	2.092683	5.912499
4	O	3.615359	4.179113	0.018647
5	O	3.615359	0.002651	2.971852
6	O	-0.002599	2.061828	5.881179
7	O	5.423820	1.046766	0.018647
8	O	1.806898	3.134997	2.971852
9	O	-1.838273	5.239659	5.880837

10	O	1.806898	1.046766	0.018647
11	O	-1.810024	3.134997	2.971852
12	O	1.832749	5.239499	5.880855
13	O	-0.001563	2.090882	1.495250
14	O	3.615359	4.179113	4.448454
15	O	3.613612	-0.034436	7.429230
16	O	3.615359	2.090882	1.495250
17	O	-0.001563	4.179113	4.448454
18	O	-0.003257	0.005502	7.470115
19	O	1.806898	5.223228	1.495250
20	O	1.806898	1.046766	4.448454
21	O	-1.776621	3.159073	7.431573
22	O	-1.810024	5.223228	1.495250
23	O	5.423820	1.046766	4.448454
24	O	1.769988	3.159045	7.430750
25	Zr	-0.001563	0.002651	0.756949
26	Zr	3.615359	2.090882	3.710153
27	Zr	-0.001059	4.174990	6.662471
28	Zr	-1.810024	3.134997	0.756949
29	Zr	1.806898	5.223228	3.710153
30	Zr	1.796194	1.035365	6.626571
31	Zr	1.806898	3.134997	0.756949
32	Zr	-1.810024	5.223228	3.710153
33	Zr	5.435591	1.035411	6.626436
34	Zr	3.615359	0.002651	0.756949
35	Zr	-0.001563	2.090882	3.710153
36	Zr	3.615920	4.188539	6.626527
37	N	3.598009	2.097440	10.077639
38	O	3.648886	2.198587	11.232416

Path1’--i

ATOM		X (Angstroms)	Y (Angstroms)	Z (Angstroms)
1	O	0.000000	4.176462	0.000000
2	O	0.000000	0.000000	2.953204
3	O	3.616261	2.088501	5.867599
4	O	3.616922	4.176462	0.000000
5	O	3.616922	0.000000	2.953204
6	O	-0.000623	2.088511	5.867624
7	O	5.425383	1.044115	0.000000
8	O	1.808461	3.132346	2.953204
9	O	-1.809087	5.220856	5.867633
10	O	1.808461	1.044115	0.000000
11	O	-1.808461	3.132346	2.953204
12	O	1.807810	5.220838	5.867621
13	O	0.000000	2.088231	1.476602
14	O	3.616922	4.176462	4.429807
15	O	3.616075	0.000860	7.421437
16	O	3.616922	2.088231	1.476602
17	O	0.000000	4.176462	4.429807
18	O	-0.000841	0.000868	7.421427
19	O	1.808461	5.220577	1.476602
20	O	1.808461	1.044115	4.429807
21	O	-1.809284	3.133196	7.421417
22	O	-1.808461	5.220577	1.476602
23	O	5.425383	1.044115	4.429807
24	O	1.807617	3.133197	7.421427
25	Zr	0.000000	0.000000	0.738301
26	Zr	3.616922	2.088231	3.691505
27	Zr	-0.000387	4.176055	6.615201
28	Zr	-1.808461	3.132346	0.738301
29	Zr	1.808461	5.220577	3.691505
30	Zr	1.808069	1.043731	6.615214
31	Zr	1.808461	3.132346	0.738301
32	Zr	-1.808461	5.220577	3.691505
33	Zr	5.424969	1.043721	6.615217
34	Zr	3.616922	0.000000	0.738301
35	Zr	0.000000	2.088231	3.691505
36	Zr	3.616519	4.176041	6.615210

Path1'--ii

ATOM		X (Angstroms)	Y (Angstroms)	Z (Angstroms)
1	O	0.000000	4.176462	0.000000
2	O	0.000000	0.000000	2.953204
3	O	3.615472	2.135226	5.903018
4	O	3.616922	4.176462	0.000000
5	O	3.616922	0.000000	2.953204
6	O	-0.001947	2.075774	5.823508
7	O	5.425383	1.044115	0.000000
8	O	1.808461	3.132346	2.953204
9	O	-1.847578	5.201149	5.895712
10	O	1.808461	1.044115	0.000000
11	O	-1.808461	3.132346	2.953204
12	O	1.845958	5.200138	5.896813
13	O	0.000000	2.088231	1.476602
14	O	3.616922	4.176462	4.429807
15	O	3.615476	0.004107	7.400433
16	O	3.616922	2.088231	1.476602
17	O	0.000000	4.176462	4.429807
18	O	-0.000989	0.067013	7.443354
19	O	1.808461	5.220577	1.476602
20	O	1.808461	1.044115	4.429807
21	O	-1.715338	3.086054	7.445289
22	O	-1.808461	5.220577	1.476602
23	O	5.425383	1.044115	4.429807
24	O	1.707506	3.082246	7.447161
25	Zr	0.000000	0.000000	0.738301
26	Zr	3.616922	2.088231	3.691505
27	Zr	0.001529	4.132668	6.626368
28	Zr	-1.808461	3.132346	0.738301
29	Zr	1.808461	5.220577	3.691505
30	Zr	1.808717	1.061464	6.616846
31	Zr	1.808461	3.132346	0.738301
32	Zr	-1.808461	5.220577	3.691505
33	Zr	5.424490	1.059670	6.615697
34	Zr	3.616922	0.000000	0.738301
35	Zr	0.000000	2.088231	3.691505
36	Zr	3.621648	4.228303	6.642459
37	C	3.602396	4.122415	9.206923
38	O	3.591244	4.084945	10.343139

Path 1'—TS1

ATOM	X (au)	Y (au)	Z (au)
O	0.00000000000000	7.89236871082582	0.00000000000000
O	0.00000000000000	0.00000000000000	5.58074743531643
O	6.52330116367135	4.06504487362176	11.33895919276046
O	6.83499179893255	7.89236871082582	0.00000000000000
O	6.83499179893255	0.00000000000000	5.58074743531643
O	0.25415935390844	4.07813192623600	11.24857605451516
O	10.25248770028855	1.97309217723402	0.00000000000000
O	3.41749589946627	5.91927653359180	5.58074743531643
O	-3.56067659669520	9.76735655752468	11.21856738884342
O	3.41749589946627	1.97309217723402	0.00000000000000
O	-3.41749589946627	5.91927653359180	5.58074743531643
O	3.37151589597516	9.55065927037839	11.31240673412612
O	0.00000000000000	3.94618435635777	2.79037371765821
O	6.83499179893255	7.89236871082582	8.37112115297464
O	6.88377154317168	0.01496932754913	14.05649462824524
O	6.83499179893255	3.94618435635777	2.79037371765821
O	0.00000000000000	7.89236871082582	8.37112115297464
O	-0.13903131030298	0.10057535720319	14.19003295067823
O	3.41749589946627	9.86546088805984	2.79037371765821
O	3.41749589946627	1.97309217723402	8.37112115297464
O	-3.37736223050518	5.72965937708518	14.16666532118166
O	-3.41749589946627	9.86546088805984	2.79037371765821
O	10.25248770028855	1.97309217723402	8.37112115297464
O	5.37823310398687	6.88841505379506	16.52250782640846

Zr	0.00000000000000	0.00000000000000	1.39518685788424
Zr	6.83499179893255	3.94618435635777	6.97593429320067
Zr	-0.17980624837596	7.95206434464241	12.45252612832283
Zr	-3.41749589946627	5.91927653359180	1.39518685788424
Zr	3.41749589946627	9.86546088805984	6.97593429320067
Zr	3.37624942181278	1.75665669841427	12.43991618431200
Zr	3.41749589946627	5.91927653359180	1.39518685788424
Zr	-3.41749589946627	9.86546088805984	6.97593429320067
Zr	10.22157603465716	1.95246943729497	12.70951230275467
Zr	6.83499179893255	0.00000000000000	1.39518685788424
Zr	0.00000000000000	3.94618435635777	6.97593429320067
Zr	6.85218975042075	7.89808509644913	12.73797420869584
C	6.14797259330503	7.22548835241716	18.72227394490814
O	5.84214485980279	7.00286833856225	20.93040723589679

Path1'--iii

ATOM		X (Angstroms)	Y (Angstroms)	Z (Angstroms)
1	O	0.000000	4.176462	0.000000
2	O	0.000000	0.000000	2.953204
3	O	3.475569	2.176712	5.927659
4	O	3.616922	4.176462	0.000000
5	O	3.616922	0.000000	2.953204
6	O	0.146285	2.167457	5.929921
7	O	5.425383	1.044115	0.000000
8	O	1.808461	3.132346	2.953204
9	O	-1.806637	5.218303	5.922346
10	O	1.808461	1.044115	0.000000
11	O	-1.808461	3.132346	2.953204
12	O	1.800900	5.047414	5.927030
13	O	0.000000	2.088231	1.476602
14	O	3.616922	4.176462	4.429807
15	O	3.692805	0.057977	7.430202
16	O	3.616922	2.088231	1.476602
17	O	0.000000	4.176462	4.429807
18	O	-0.084309	0.034133	7.428039
19	O	1.808461	5.220577	1.476602
20	O	1.808461	1.044115	4.429807
21	O	-1.801260	3.036436	7.437936
22	O	-1.808461	5.220577	1.476602
23	O	5.425383	1.044115	4.429807
24	O	2.421021	3.133512	10.467484
25	Zr	0.000000	0.000000	0.738301
26	Zr	3.616922	2.088231	3.691505
27	Zr	-0.041613	4.166917	6.580606
28	Zr	-1.808461	3.132346	0.738301
29	Zr	1.808461	5.220577	3.691505
30	Zr	1.838696	1.008725	6.579854
31	Zr	1.808461	3.132346	0.738301
32	Zr	-1.808461	5.220577	3.691505
33	Zr	5.421164	1.054087	6.734440
34	Zr	3.616922	0.000000	0.738301
35	Zr	0.000000	2.088231	3.691505
36	Zr	3.622957	4.223550	6.580151
37	C	3.047176	3.365115	11.434667
38	O	3.674233	3.599149	12.399805

Path1'--iv

ATOM		X (Angstroms)	Y (Angstroms)	Z (Angstroms)
1	O	0.000000	4.176462	0.000000
2	O	0.000000	0.000000	2.953204
3	O	3.470638	2.180833	5.925438
4	O	3.616922	4.176462	0.000000
5	O	3.616922	0.000000	2.953204
6	O	0.149708	2.175360	5.928687
7	O	5.425383	1.044115	0.000000
8	O	1.808461	3.132346	2.953204
9	O	-1.806261	5.214904	5.925289
10	O	1.808461	1.044115	0.000000

11	O	-1.808461	3.132346	2.953204
12	O	1.801979	5.041870	5.937327
13	O	0.000000	2.088231	1.476602
14	O	3.616922	4.176462	4.429807
15	O	3.692572	0.065282	7.428560
16	O	3.616922	2.088231	1.476602
17	O	0.000000	4.176462	4.429807
18	O	-0.083563	0.044570	7.426353
19	O	1.808461	5.220577	1.476602
20	O	1.808461	1.044115	4.429807
21	O	-1.803339	3.032930	7.441990
22	O	-1.808461	5.220577	1.476602
23	O	5.425383	1.044115	4.429807
24	Zr	0.000000	0.000000	0.738301
25	Zr	3.616922	2.088231	3.691505
26	Zr	-0.040440	4.169275	6.582316
27	Zr	-1.808461	3.132346	0.738301
28	Zr	1.808461	5.220577	3.691505
29	Zr	1.831660	1.014107	6.581435
30	Zr	1.808461	3.132346	0.738301
31	Zr	-1.808461	5.220577	3.691505
32	Zr	5.421764	1.047844	6.743144
33	Zr	3.616922	0.000000	0.738301
34	Zr	0.000000	2.088231	3.691505
35	Zr	3.632057	4.215216	6.579495

Path1’--v

ATOM		X (Angstroms)	Y (Angstroms)	Z (Angstroms)
1	O	0.000000	4.176462	0.000000
2	O	0.000000	0.000000	2.953204
3	O	3.495039	2.163517	5.871498
4	O	3.616922	4.176462	0.000000
5	O	3.616922	0.000000	2.953204
6	O	0.121082	2.155564	5.869250
7	O	5.425383	1.044115	0.000000
8	O	1.808461	3.132346	2.953204
9	O	-1.813476	5.220163	5.951731
10	O	1.808461	1.044115	0.000000
11	O	-1.808461	3.132346	2.953204
12	O	1.806101	5.079289	5.871095
13	O	0.000000	2.088231	1.476602
14	O	3.616922	4.176462	4.429807
15	O	3.695182	0.056614	7.398066
16	O	3.616922	2.088231	1.476602
17	O	0.000000	4.176462	4.429807
18	O	-0.088727	0.040796	7.402231
19	O	1.808461	5.220577	1.476602
20	O	1.808461	1.044115	4.429807
21	O	-1.796645	3.035685	7.401368
22	O	-1.808461	5.220577	1.476602
23	O	5.425383	1.044115	4.429807
24	Zr	0.000000	0.000000	0.738301
25	Zr	3.616922	2.088231	3.691505
26	Zr	-0.015466	4.159244	6.610661
27	Zr	-1.808461	3.132346	0.738301
28	Zr	1.808461	5.220577	3.691505
29	Zr	1.827485	1.029068	6.613543
30	Zr	1.808461	3.132346	0.738301
31	Zr	-1.808461	5.220577	3.691505
32	Zr	5.422899	1.045903	6.695898
33	Zr	3.616922	0.000000	0.738301
34	Zr	0.000000	2.088231	3.691505
35	Zr	3.620497	4.202313	6.607914
36	N	1.802943	3.130353	7.809257
37	O	1.771842	3.194638	9.092862

Path1’--vi

ATOM		X (Angstroms)	Y (Angstroms)	Z (Angstroms)

1	O	0.000000	4.176462	0.000000
2	O	0.000000	0.000000	2.953204
3	O	3.517598	2.145528	5.877831
4	O	3.616922	4.176462	0.000000
5	O	3.616922	0.000000	2.953204
6	O	0.109076	2.150181	5.864237
7	O	5.425383	1.044115	0.000000
8	O	1.808461	3.132346	2.953204
9	O	-1.808061	5.220841	5.950855
10	O	1.808461	1.044115	0.000000
11	O	-1.808461	3.132346	2.953204
12	O	1.802587	5.106581	5.878836
13	O	0.000000	2.088231	1.476602
14	O	3.616922	4.176462	4.429807
15	O	3.683787	0.049848	7.420395
16	O	3.616922	2.088231	1.476602
17	O	0.000000	4.176462	4.429807
18	O	7.151767	0.046702	7.401588
19	O	1.808461	5.220577	1.476602
20	O	1.808461	1.044115	4.429807
21	O	5.434558	3.037669	7.401444
22	O	-1.808461	5.220577	1.476602
23	O	5.425383	1.044115	4.429807
24	Zr	0.000000	0.000000	0.738301
25	Zr	3.616922	2.088231	3.691505
26	Zr	-0.025094	4.174109	6.612864
27	Zr	-1.808461	3.132346	0.738301
28	Zr	1.808461	5.220577	3.691505
29	Zr	1.821913	1.023510	6.613698
30	Zr	1.808461	3.132346	0.738301
31	Zr	-1.808461	5.220577	3.691505
32	Zr	5.422007	1.044354	6.678509
33	Zr	3.616922	0.000000	0.738301
34	Zr	0.000000	2.088231	3.691505
35	Zr	3.601807	4.185746	6.660132
36	N	1.741639	3.093710	7.695991
37	O	2.009237	3.241719	9.169340
38	C	3.149627	3.891711	9.322417
39	O	3.722129	4.209601	10.321088

Path 1’—TS2

ATOM		X (Angstroms)	Y (Angstroms)	Z (Angstroms)
1	O	0.000000	4.176462	0.000000
2	O	0.000000	0.000000	2.953204
3	O	3.528651	2.139937	5.867021
4	O	3.616922	4.176462	0.000000
5	O	3.616922	0.000000	2.953204
6	O	0.086260	2.137674	5.862344
7	O	5.425383	1.044115	0.000000
8	O	1.808461	3.132346	2.953204
9	O	-1.809991	5.219470	5.934654
10	O	1.808461	1.044115	0.000000
11	O	-1.808461	3.132346	2.953204
12	O	1.802911	5.115710	5.871277
13	O	0.000000	2.088231	1.476602
14	O	3.616922	4.176462	4.429807
15	O	3.678468	0.046439	7.415547
16	O	3.616922	2.088231	1.476602
17	O	0.000000	4.176462	4.429807
18	O	-0.076446	0.036960	7.407120
19	O	1.808461	5.220577	1.476602
20	O	1.808461	1.044115	4.429807
21	O	-1.805258	3.048389	7.407148
22	O	-1.808461	5.220577	1.476602
23	O	5.425383	1.044115	4.429807
24	Zr	0.000000	0.000000	0.738301
25	Zr	3.616922	2.088231	3.691505
26	Zr	-0.018740	4.169101	6.613973

27	Zr	-1.808461	3.132346	0.738301
28	Zr	1.808461	5.220577	3.691505
29	Zr	1.819660	1.035458	6.612862
30	Zr	1.808461	3.132346	0.738301
31	Zr	-1.808461	5.220577	3.691505
32	Zr	5.421046	1.042518	6.673631
33	Zr	3.616922	0.000000	0.738301
34	Zr	0.000000	2.088231	3.691505
35	Zr	3.618316	4.192659	6.633785
36	N	1.768406	3.110848	7.647298
37	O	1.938472	3.188303	9.369149
38	C	3.019862	3.781580	9.613854
39	O	3.710491	4.141610	10.519226

Path1’--vii

ATOM		X (Angstroms)	Y (Angstroms)	Z (Angstroms)
1	O	0.000000	4.176462	0.000000
2	O	0.000000	0.000000	2.953204
3	O	3.567271	2.112296	5.858675
4	O	3.616922	4.176462	0.000000
5	O	3.616922	0.000000	2.953204
6	O	0.050018	2.116152	5.863586
7	O	5.425383	1.044115	0.000000
8	O	1.808461	3.132346	2.953204
9	O	-1.800729	5.225698	5.908447
10	O	1.808461	1.044115	0.000000
11	O	-1.808461	3.132346	2.953204
12	O	1.800118	5.166297	5.860546
13	O	0.000000	2.088231	1.476602
14	O	3.616922	4.176462	4.429807
15	O	3.667698	0.041211	7.437019
16	O	3.616922	2.088231	1.476602
17	O	0.000000	4.176462	4.429807
18	O	-0.057274	0.016763	7.414499
19	O	1.808461	5.220577	1.476602
20	O	1.808461	1.044115	4.429807
21	O	-1.814719	3.077739	7.412828
22	O	-1.808461	5.220577	1.476602
23	O	5.425383	1.044115	4.429807
24	Zr	0.000000	0.000000	0.738301
25	Zr	3.616922	2.088231	3.691505
26	Zr	0.004091	4.158998	6.622784
27	Zr	-1.808461	3.132346	0.738301
28	Zr	1.808461	5.220577	3.691505
29	Zr	1.821025	1.055872	6.622606
30	Zr	1.808461	3.132346	0.738301
31	Zr	-1.808461	5.220577	3.691505
32	Zr	5.418676	1.039410	6.683997
33	Zr	3.616922	0.000000	0.738301
34	Zr	0.000000	2.088231	3.691505
35	Zr	3.606752	4.181814	6.633496
36	N	1.811426	3.136402	7.540019
37	O	0.438890	2.359109	10.438600
38	C	1.446733	2.906282	10.698693
39	O	2.449830	3.452083	10.973597

Path1’--viii

ATOM		X (Angstroms)	Y (Angstroms)	Z (Angstroms)
1	O	0.000000	4.176462	0.000000
2	O	0.000000	0.000000	2.953204
3	O	3.567385	2.112517	5.858394
4	O	3.616922	4.176462	0.000000
5	O	3.616922	0.000000	2.953204
6	O	0.050073	2.116140	5.863583
7	O	5.425383	1.044115	0.000000
8	O	1.808461	3.132346	2.953204
9	O	-1.800403	5.225873	5.908753
10	O	1.808461	1.044115	0.000000

11	O	-1.808461	3.132346	2.953204
12	O	1.800208	5.166321	5.860188
13	O	0.000000	2.088231	1.476602
14	O	3.616922	4.176462	4.429807
15	O	3.668493	0.041463	7.436126
16	O	3.616922	2.088231	1.476602
17	O	0.000000	4.176462	4.429807
18	O	-0.057433	0.017557	7.417032
19	O	1.808461	5.220577	1.476602
20	O	1.808461	1.044115	4.429807
21	O	-1.814348	3.077220	7.415468
22	O	-1.808461	5.220577	1.476602
23	O	5.425383	1.044115	4.429807
24	Zr	0.000000	0.000000	0.738301
25	Zr	3.616922	2.088231	3.691505
26	Zr	0.004041	4.159103	6.622803
27	Zr	-1.808461	3.132346	0.738301
28	Zr	1.808461	5.220577	3.691505
29	Zr	1.821231	1.055748	6.622679
30	Zr	1.808461	3.132346	0.738301
31	Zr	-1.808461	5.220577	3.691505
32	Zr	5.418797	1.039422	6.683272
33	Zr	3.616922	0.000000	0.738301
34	Zr	0.000000	2.088231	3.691505
35	Zr	3.607370	4.182254	6.633290
36	N	1.811795	3.136640	7.538386

Path1’--ix

ATOM		X (Angstroms)	Y (Angstroms)	Z (Angstroms)
1	O	0.000000	4.176462	0.000000
2	O	0.000000	0.000000	2.953204
3	O	3.566484	2.121439	5.859590
4	O	3.616922	4.176462	0.000000
5	O	3.616922	0.000000	2.953204
6	O	0.052598	2.120940	5.866365
7	O	5.425383	1.044115	0.000000
8	O	1.808461	3.132346	2.953204
9	O	-1.815051	5.217763	5.922650
10	O	1.808461	1.044115	0.000000
11	O	-1.808461	3.132346	2.953204
12	O	1.804068	5.153184	5.865651
13	O	0.000000	2.088231	1.476602
14	O	3.616922	4.176462	4.429807
15	O	3.672470	0.041267	7.444814
16	O	3.616922	2.088231	1.476602
17	O	0.000000	4.176462	4.429807
18	O	-0.072621	0.031924	7.450962
19	O	1.808461	5.220577	1.476602
20	O	1.808461	1.044115	4.429807
21	O	-1.808941	3.063386	7.449588
22	O	-1.808461	5.220577	1.476602
23	O	5.425383	1.044115	4.429807
24	Zr	0.000000	0.000000	0.738301
25	Zr	3.616922	2.088231	3.691505
26	Zr	-0.003378	4.169819	6.614033
27	Zr	-1.808461	3.132346	0.738301
28	Zr	1.808461	5.220577	3.691505
29	Zr	1.827640	1.080093	6.625018
30	Zr	1.808461	3.132346	0.738301
31	Zr	-1.808461	5.220577	3.691505
32	Zr	5.440580	1.036710	6.680509
33	Zr	3.616922	0.000000	0.738301
34	Zr	0.000000	2.088231	3.691505
35	Zr	3.600400	4.165951	6.613126
36	N	1.798231	3.126007	7.628807
37	O	2.013312	3.078070	9.674567
38	N	1.365803	3.715679	10.359339

Path 1'—TS3

ATOM		X (Angstroms)	Y (Angstroms)	Z (Angstroms)
1	O	0.000000	4.176462	0.000000
2	O	0.000000	0.000000	2.953204
3	O	3.586368	2.111421	5.865791
4	O	3.616922	4.176462	0.000000
5	O	3.616922	0.000000	2.953204
6	O	0.030820	2.107094	5.863554
7	O	5.425383	1.044115	0.000000
8	O	1.808461	3.132346	2.953204
9	O	-1.814045	5.210001	5.914074
10	O	1.808461	1.044115	0.000000
11	O	-1.808461	3.132346	2.953204
12	O	1.809632	5.177340	5.864637
13	O	0.000000	2.088231	1.476602
14	O	3.616922	4.176462	4.429807
15	O	3.666392	0.026367	7.454588
16	O	3.616922	2.088231	1.476602
17	O	0.000000	4.176462	4.429807
18	O	-0.058894	0.026994	7.459441
19	O	1.808461	5.220577	1.476602
20	O	1.808461	1.044115	4.429807
21	O	-1.803226	3.071593	7.460373
22	O	-1.808461	5.220577	1.476602
23	O	5.425383	1.044115	4.429807
24	Zr	0.000000	0.000000	0.738301
25	Zr	3.616922	2.088231	3.691505
26	Zr	0.015658	4.162248	6.630608
27	Zr	-1.808461	3.132346	0.738301
28	Zr	1.808461	5.220577	3.691505
29	Zr	1.815062	1.087992	6.636756
30	Zr	1.808461	3.132346	0.738301
31	Zr	-1.808461	5.220577	3.691505
32	Zr	5.429409	1.041600	6.672788
33	Zr	3.616922	0.000000	0.738301
34	Zr	0.000000	2.088231	3.691505
35	Zr	3.610582	4.179759	6.612705
36	N	1.808881	3.114633	7.567526
37	O	2.459942	3.223732	10.183154
38	N	1.468754	3.731725	10.393050

Path1'--x

ATOM		X (Angstroms)	Y (Angstroms)	Z (Angstroms)
1	O	0.000000	4.176462	0.000000
2	O	0.000000	0.000000	2.953204
3	O	3.533236	2.149329	5.910255
4	O	3.616922	4.176462	0.000000
5	O	3.616922	0.000000	2.953204
6	O	0.093194	2.139149	5.839670
7	O	5.425383	1.044115	0.000000
8	O	1.808461	3.132346	2.953204
9	O	-1.804232	5.225323	5.928037
10	O	1.808461	1.044115	0.000000
11	O	-1.808461	3.132346	2.953204
12	O	1.817302	5.123647	5.908834
13	O	0.000000	2.088231	1.476602
14	O	3.616922	4.176462	4.429807
15	O	3.660321	0.033380	7.405188
16	O	3.616922	2.088231	1.476602
17	O	0.000000	4.176462	4.429807
18	O	-0.035595	0.064549	7.367630
19	O	1.808461	5.220577	1.476602
20	O	1.808461	1.044115	4.429807
21	O	-1.764302	3.071538	7.359069
22	O	-1.808461	5.220577	1.476602
23	O	5.425383	1.044115	4.429807
24	Zr	0.000000	0.000000	0.738301
25	Zr	3.616922	2.088231	3.691505

26	Zr	-0.045005	4.156037	6.603905
27	Zr	-1.808461	3.132346	0.738301
28	Zr	1.808461	5.220577	3.691505
29	Zr	1.782670	1.012683	6.603245
30	Zr	1.808461	3.132346	0.738301
31	Zr	-1.808461	5.220577	3.691505
32	Zr	5.395167	1.027282	6.650448
33	Zr	3.616922	0.000000	0.738301
34	Zr	0.000000	2.088231	3.691505
35	Zr	3.660316	4.210629	6.690696
36	N	1.652359	3.050701	7.755803
37	N	2.096822	3.285497	8.964544
38	O	3.218522	3.908571	8.961052

Path1'--xi

ATOM		X (Angstroms)	Y (Angstroms)	Z (Angstroms)
1	O	0.000000	4.176462	0.000000
2	O	0.000000	0.000000	2.953204
3	O	3.592842	2.094608	5.860185
4	O	3.616922	4.176462	0.000000
5	O	3.616922	0.000000	2.953204
6	O	0.029138	2.102211	5.872530
7	O	5.425383	1.044115	0.000000
8	O	1.808461	3.132346	2.953204
9	O	-1.792992	5.233323	5.886652
10	O	1.808461	1.044115	0.000000
11	O	-1.808461	3.132346	2.953204
12	O	1.798983	5.200389	5.859423
13	O	0.000000	2.088231	1.476602
14	O	3.616922	4.176462	4.429807
15	O	3.652478	0.030438	7.430453
16	O	3.616922	2.088231	1.476602
17	O	0.000000	4.176462	4.429807
18	O	-0.025401	0.001439	7.412092
19	O	1.808461	5.220577	1.476602
20	O	1.808461	1.044115	4.429807
21	O	-1.810884	3.116142	7.406782
22	O	-1.808461	5.220577	1.476602
23	O	5.425383	1.044115	4.429807
24	Zr	0.000000	0.000000	0.738301
25	Zr	3.616922	2.088231	3.691505
26	Zr	0.001450	4.154295	6.604642
27	Zr	-1.808461	3.132346	0.738301
28	Zr	1.808461	5.220577	3.691505
29	Zr	1.814094	1.048562	6.600703
30	Zr	1.808461	3.132346	0.738301
31	Zr	-1.808461	5.220577	3.691505
32	Zr	5.408579	1.031013	6.644068
33	Zr	3.616922	0.000000	0.738301
34	Zr	0.000000	2.088231	3.691505
35	Zr	3.594395	4.172224	6.617536
36	N	1.116794	3.265023	10.640083
37	N	2.079911	3.519283	11.126536
38	O	1.825747	3.147913	7.442135

Path2'--v

ATOM		X (Angstroms)	Y (Angstroms)	Z (Angstroms)
1	O	0.000000	4.176462	0.000000
2	O	0.000000	0.000000	2.953204
3	O	3.528945	2.142296	5.921004
4	O	3.616922	4.176462	0.000000
5	O	3.616922	0.000000	2.953204
6	O	0.086301	2.142170	5.843336
7	O	5.425383	1.044115	0.000000
8	O	1.808461	3.132346	2.953204
9	O	-1.777223	5.232984	5.909533
10	O	1.808461	1.044115	0.000000
11	O	-1.808461	3.132346	2.953204
12	O	1.804701	5.117970	5.926620

13	O	0.000000	2.088231	1.476602
14	O	3.616922	4.176462	4.429807
15	O	3.648894	0.029752	7.410918
16	O	3.616922	2.088231	1.476602
17	O	0.000000	4.176462	4.429807
18	O	-0.010285	0.091921	7.374388
19	O	1.808461	5.220577	1.476602
20	O	1.808461	1.044115	4.429807
21	O	-1.733333	3.075115	7.380888
22	O	-1.808461	5.220577	1.476602
23	O	5.425383	1.044115	4.429807
24	Zr	0.000000	0.000000	0.738301
25	Zr	3.616922	2.088231	3.691505
26	Zr	-0.042852	4.185447	6.583635
27	Zr	-1.808461	3.132346	0.738301
28	Zr	1.808461	5.220577	3.691505
29	Zr	1.820203	0.990328	6.590020
30	Zr	1.808461	3.132346	0.738301
31	Zr	-1.808461	5.220577	3.691505
32	Zr	5.427932	1.047031	6.665408
33	Zr	3.616922	0.000000	0.738301
34	Zr	0.000000	2.088231	3.691505
35	Zr	3.694834	4.227651	6.673618
36	O	1.524933	2.960139	7.761056
37	N	2.586797	3.560585	8.403537

Path2'--vi

ATOM		X (Angstroms)	Y (Angstroms)	Z (Angstroms)
1	O	0.000000	4.176462	0.000000
2	O	0.000000	0.000000	2.953204
3	O	3.524767	2.141024	5.954596
4	O	3.616922	4.176462	0.000000
5	O	3.616922	0.000000	2.953204
6	O	0.082117	2.122081	5.855258
7	O	5.425383	1.044115	0.000000
8	O	1.808461	3.132346	2.953204
9	O	-1.791910	5.244617	5.882363
10	O	1.808461	1.044115	0.000000
11	O	-1.808461	3.132346	2.953204
12	O	1.818597	5.131798	5.943100
13	O	0.000000	2.088231	1.476602
14	O	3.616922	4.176462	4.429807
15	O	3.603756	-0.026133	7.410861
16	O	3.616922	2.088231	1.476602
17	O	0.000000	4.176462	4.429807
18	O	0.027557	0.085937	7.398947
19	O	1.808461	5.220577	1.476602
20	O	1.808461	1.044115	4.429807
21	O	-1.715733	3.133505	7.376823
22	O	-1.808461	5.220577	1.476602
23	O	5.425383	1.044115	4.429807
24	Zr	0.000000	0.000000	0.738301
25	Zr	3.616922	2.088231	3.691505
26	Zr	-0.062760	4.218254	6.584864
27	Zr	-1.808461	3.132346	0.738301
28	Zr	1.808461	5.220577	3.691505
29	Zr	1.813641	0.985235	6.571296
30	Zr	1.808461	3.132346	0.738301
31	Zr	-1.808461	5.220577	3.691505
32	Zr	5.464356	1.031422	6.735676
33	Zr	3.616922	0.000000	0.738301
34	Zr	0.000000	2.088231	3.691505
35	Zr	3.693269	4.215258	6.678548
36	O	1.460626	2.945846	7.832142
37	N	2.551196	3.570763	8.343427
38	C	5.304357	1.076614	9.264795
39	O	5.181822	1.105505	10.393508

Path2'—TS2

ATOM		X (Angstroms)	Y (Angstroms)	Z (Angstroms)
1	O	0.000000	4.176462	0.000000
2	O	0.000000	0.000000	2.953204
3	O	3.508111	2.155190	5.921663
4	O	3.616922	4.176462	0.000000
5	O	3.616922	0.000000	2.953204
6	O	0.102292	2.152277	5.833348
7	O	5.425383	1.044115	0.000000
8	O	1.808461	3.132346	2.953204
9	O	-1.771913	5.234840	5.916572
10	O	1.808461	1.044115	0.000000
11	O	-1.808461	3.132346	2.953204
12	O	1.813473	5.088269	5.922475
13	O	0.000000	2.088231	1.476602
14	O	3.616922	4.176462	4.429807
15	O	3.670594	0.028702	7.402289
16	O	3.616922	2.088231	1.476602
17	O	0.000000	4.176462	4.429807
18	O	-0.008538	0.107428	7.362165
19	O	1.808461	5.220577	1.476602
20	O	1.808461	1.044115	4.429807
21	O	-1.727126	3.067382	7.369432
22	O	-1.808461	5.220577	1.476602
23	O	5.425383	1.044115	4.429807
24	Zr	0.000000	0.000000	0.738301
25	Zr	3.616922	2.088231	3.691505
26	Zr	-0.043324	4.200492	6.599033
27	Zr	-1.808461	3.132346	0.738301
28	Zr	1.808461	5.220577	3.691505
29	Zr	1.799058	0.993941	6.584308
30	Zr	1.808461	3.132346	0.738301
31	Zr	-1.808461	5.220577	3.691505
32	Zr	5.420352	1.048930	6.668394
33	Zr	3.616922	0.000000	0.738301
34	Zr	0.000000	2.088231	3.691505
35	Zr	3.697848	4.225734	6.669654
36	O	1.409212	2.892933	7.850905
37	N	2.512398	3.565001	8.287562
38	C	2.934566	3.252909	10.312500
39	O	2.110917	2.660189	10.862898

Path2'—vii

ATOM		X (Angstroms)	Y (Angstroms)	Z (Angstroms)
1	O	0.000000	4.176462	0.000000
2	O	0.000000	0.000000	2.953204
3	O	3.632938	2.127791	5.919623
4	O	3.616922	4.176462	0.000000
5	O	3.616922	0.000000	2.953204
6	O	-0.009887	2.077705	5.807281
7	O	5.425383	1.044115	0.000000
8	O	1.808461	3.132346	2.953204
9	O	-1.848819	5.200918	5.899365
10	O	1.808461	1.044115	0.000000
11	O	-1.808461	3.132346	2.953204
12	O	1.852467	5.219096	5.910285
13	O	0.000000	2.088231	1.476602
14	O	3.616922	4.176462	4.429807
15	O	3.599764	-0.009859	7.393407
16	O	3.616922	2.088231	1.476602
17	O	0.000000	4.176462	4.429807
18	O	0.012126	0.096666	7.415464
19	O	1.808461	5.220577	1.476602
20	O	1.808461	1.044115	4.429807
21	O	-1.679118	3.086207	7.414396
22	O	-1.808461	5.220577	1.476602
23	O	5.425383	1.044115	4.429807
24	Zr	0.000000	0.000000	0.738301

25	Zr	3.616922	2.088231	3.691505
26	Zr	-0.004727	4.175924	6.616009
27	Zr	-1.808461	3.132346	0.738301
28	Zr	1.808461	5.220577	3.691505
29	Zr	1.807503	1.023482	6.610193
30	Zr	1.808461	3.132346	0.738301
31	Zr	-1.808461	5.220577	3.691505
32	Zr	5.435366	1.043362	6.591839
33	Zr	3.616922	0.000000	0.738301
34	Zr	0.000000	2.088231	3.691505
35	Zr	3.605430	4.197944	6.808300
36	O	1.695986	3.086774	7.390993
37	N	3.658439	4.012738	9.019417
38	C	3.782670	3.477921	10.101825
39	O	3.900064	2.973575	11.173555

Path2'—viii

ATOM		X (Angstroms)	Y (Angstroms)	Z (Angstroms)
1	O	0.000000	4.176462	0.000000
2	O	0.000000	0.000000	2.953204
3	O	3.527809	2.181888	5.949075
4	O	3.616922	4.176462	0.000000
5	O	3.616922	0.000000	2.953204
6	O	0.082890	2.135162	5.801397
7	O	5.425383	1.044115	0.000000
8	O	1.808461	3.132346	2.953204
9	O	-1.858829	5.197306	5.955038
10	O	1.808461	1.044115	0.000000
11	O	-1.808461	3.132346	2.953204
12	O	1.844701	5.098450	5.946169
13	O	0.000000	2.088231	1.476602
14	O	3.616922	4.176462	4.429807
15	O	3.646821	0.016748	7.374173
16	O	3.616922	2.088231	1.476602
17	O	0.000000	4.176462	4.429807
18	O	-0.033758	0.152334	7.384749
19	O	1.808461	5.220577	1.476602
20	O	1.808461	1.044115	4.429807
21	O	-1.688396	3.031972	7.379659
22	O	-1.808461	5.220577	1.476602
23	O	5.425383	1.044115	4.429807
24	Zr	0.000000	0.000000	0.738301
25	Zr	3.616922	2.088231	3.691505
26	Zr	-0.047711	4.178575	6.577124
27	Zr	-1.808461	3.132346	0.738301
28	Zr	1.808461	5.220577	3.691505
29	Zr	1.789146	1.015230	6.577852
30	Zr	1.808461	3.132346	0.738301
31	Zr	-1.808461	5.220577	3.691505
32	Zr	5.439072	1.045372	6.639065
33	Zr	3.616922	0.000000	0.738301
34	Zr	0.000000	2.088231	3.691505
35	Zr	3.618614	4.181307	6.829336
36	O	1.632632	3.039187	7.646458
37	N	3.644641	4.180467	9.060197
38	C	4.413628	4.158185	10.002007
39	O	5.140998	4.139111	10.942594
40	N	1.813686	2.916816	9.494464
41	O	1.011044	3.529010	10.017810

Path 2'—TS3

ATOM	X (au)	Y (au)	Z (au)
O	0.00000000000000	7.89236871082582	0.00000000000000
O	0.00000000000000	0.00000000000000	5.58074743531643
O	6.81546156446842	4.05704487113941	11.14892358285963
O	6.83499179893255	7.89236871082582	0.00000000000000
O	6.83499179893255	0.00000000000000	5.58074743531643
O	0.01930738662112	3.95399185810894	10.97908338534975

O	10.25248770028855	1.97309217723402	0.00000000000000
O	3.41749589946627	5.91927653359180	5.58074743531643
O	-3.50976422849387	9.82412336529138	11.16924022874040
O	3.41749589946627	1.97309217723402	0.00000000000000
O	-3.41749589946627	5.91927653359180	5.58074743531643
O	3.51176834730011	9.80002284898093	11.14781652838327
O	0.00000000000000	3.94618435635777	2.79037371765821
O	6.83499179893255	7.89236871082582	8.37112115297464
O	6.86127826349698	0.01675668532021	13.96128662613980
O	6.83499179893255	3.94618435635777	2.79037371765821
O	0.00000000000000	7.89236871082582	8.37112115297464
O	-0.01160238550469	0.22059231315635	14.07595112937543
O	3.41749589946627	9.86546088805984	2.79037371765821
O	3.41749589946627	1.97309217723402	8.37112115297464
O	-3.24849481027576	5.81484073047690	14.04336292518211
O	-3.41749589946627	9.86546088805984	2.79037371765821
O	10.25248770028855	1.97309217723402	8.37112115297464
Zr	0.00000000000000	0.00000000000000	1.39518685788424
Zr	6.83499179893255	3.94618435635777	6.97593429320067
Zr	0.02295370010669	7.82514484843345	12.50115854951326
Zr	-3.41749589946627	5.91927653359180	1.39518685788424
Zr	3.41749589946627	9.86546088805984	6.97593429320067
Zr	3.37013723116287	2.02197616065593	12.50432324361917
Zr	3.41749589946627	5.91927653359180	1.39518685788424
Zr	-3.41749589946627	9.86546088805984	6.97593429320067
Zr	10.29051405816888	1.98352409472279	12.51651280293569
Zr	6.83499179893255	0.00000000000000	1.39518685788424
Zr	0.00000000000000	3.94618435635777	6.97593429320067
Zr	6.82932246182057	7.85856083770719	12.53859857073044
O	3.24834309361381	5.82995115064770	14.06644215979529
N	6.82186268668394	7.92092168266896	17.48763086945845
C	8.42736995090568	9.10688978974451	19.02346819656354
O	10.24960411617181	9.91917719787036	19.86866333511428
N	4.89488209457111	7.29944913379856	18.99216608894829
O	5.26642210987669	8.15388937380143	21.17489716169009

Path2’—ix

ATOM		X (Angstroms)	Y (Angstroms)	Z (Angstroms)
1	O	0.000000	4.176462	0.000000
2	O	0.000000	0.000000	2.953204
3	O	3.619500	2.141079	5.895541
4	O	3.616922	4.176462	0.000000
5	O	3.616922	0.000000	2.953204
6	O	-0.004271	2.086965	5.822678
7	O	5.425383	1.044115	0.000000
8	O	1.808461	3.132346	2.953204
9	O	-1.855428	5.200278	5.900776
10	O	1.808461	1.044115	0.000000
11	O	-1.808461	3.132346	2.953204
12	O	1.856905	5.200942	5.897514
13	O	0.000000	2.088231	1.476602
14	O	3.616922	4.176462	4.429807
15	O	3.625692	0.007979	7.379092
16	O	3.616922	2.088231	1.476602
17	O	0.000000	4.176462	4.429807
18	O	-0.002245	0.110587	7.461274
19	O	1.808461	5.220577	1.476602
20	O	1.808461	1.044115	4.429807
21	O	-1.733781	3.093034	7.446126
22	O	-1.808461	5.220577	1.476602
23	O	5.425383	1.044115	4.429807
24	Zr	0.000000	0.000000	0.738301
25	Zr	3.616922	2.088231	3.691505
26	Zr	0.001348	4.151765	6.619225
27	Zr	-1.808461	3.132346	0.738301
28	Zr	1.808461	5.220577	3.691505
29	Zr	1.787410	1.058545	6.624054
30	Zr	1.808461	3.132346	0.738301

31	Zr	-1.808461	5.220577	3.691505
32	Zr	5.437303	1.047702	6.622188
33	Zr	3.616922	0.000000	0.738301
34	Zr	0.000000	2.088231	3.691505
35	Zr	3.621150	4.162605	6.636113
36	O	1.727872	3.086341	7.427036
37	N	3.614818	4.331908	9.200101
38	C	4.463002	5.011377	10.272201
39	O	5.489976	5.591120	10.287384
40	N	2.782305	4.008565	10.049548
41	O	3.569770	4.675742	11.246591

Path2’—TS4

ATOM		X (Angstroms)	Y (Angstroms)	Z (Angstroms)
1	O	0.000000	4.176462	0.000000
2	O	0.000000	0.000000	2.953204
3	O	3.615957	2.140917	5.896927
4	O	3.616922	4.176462	0.000000
5	O	3.616922	0.000000	2.953204
6	O	-0.001021	2.086857	5.824432
7	O	5.425383	1.044115	0.000000
8	O	1.808461	3.132346	2.953204
9	O	-1.852613	5.205033	5.896742
10	O	1.808461	1.044115	0.000000
11	O	-1.808461	3.132346	2.953204
12	O	1.858173	5.200740	5.896839
13	O	0.000000	2.088231	1.476602
14	O	3.616922	4.176462	4.429807
15	O	3.627061	0.005820	7.381711
16	O	3.616922	2.088231	1.476602
17	O	0.000000	4.176462	4.429807
18	O	0.001102	0.106835	7.458564
19	O	1.808461	5.220577	1.476602
20	O	1.808461	1.044115	4.429807
21	O	-1.733547	3.098071	7.439286
22	O	-1.808461	5.220577	1.476602
23	O	5.425383	1.044115	4.429807
24	Zr	0.000000	0.000000	0.738301
25	Zr	3.616922	2.088231	3.691505
26	Zr	0.002751	4.150816	6.623919
27	Zr	-1.808461	3.132346	0.738301
28	Zr	1.808461	5.220577	3.691505
29	Zr	1.785156	1.057007	6.625626
30	Zr	1.808461	3.132346	0.738301
31	Zr	-1.808461	5.220577	3.691505
32	Zr	5.438627	1.047684	6.621963
33	Zr	3.616922	0.000000	0.738301
34	Zr	0.000000	2.088231	3.691505
35	Zr	3.627275	4.165964	6.627900
36	O	1.730971	3.093204	7.438916
37	N	3.626076	4.356615	9.242061
38	C	4.415198	5.012714	10.418844
39	O	5.456519	5.568612	10.461138
40	N	2.733690	4.024880	9.993919
41	O	3.488299	4.695457	11.325636

Path2’—x

ATOM		X (Angstroms)	Y (Angstroms)	Z (Angstroms)
1	O	0.000000	4.176462	0.000000
2	O	0.000000	0.000000	2.953204
3	O	3.624902	2.106526	5.875204
4	O	3.616922	4.176462	0.000000
5	O	3.616922	0.000000	2.953204
6	O	-0.020976	2.083270	5.852039
7	O	5.425383	1.044115	0.000000
8	O	1.808461	3.132346	2.953204
9	O	-1.824716	5.212419	5.863846
10	O	1.808461	1.044115	0.000000

11	O	-1.808461	3.132346	2.953204
12	O	1.825816	5.216578	5.878533
13	O	0.000000	2.088231	1.476602
14	O	3.616922	4.176462	4.429807
15	O	3.609760	0.000116	7.410359
16	O	3.616922	2.088231	1.476602
17	O	0.000000	4.176462	4.429807
18	O	0.015745	0.016118	7.424025
19	O	1.808461	5.220577	1.476602
20	O	1.808461	1.044115	4.429807
21	O	-1.797525	3.149306	7.426676
22	O	-1.808461	5.220577	1.476602
23	O	5.425383	1.044115	4.429807
24	Zr	0.000000	0.000000	0.738301
25	Zr	3.616922	2.088231	3.691505
26	Zr	0.001597	4.163116	6.620292
27	Zr	-1.808461	3.132346	0.738301
28	Zr	1.808461	5.220577	3.691505
29	Zr	1.793030	1.058486	6.623746
30	Zr	1.808461	3.132346	0.738301
31	Zr	-1.808461	5.220577	3.691505
32	Zr	5.423442	1.030089	6.601025
33	Zr	3.616922	0.000000	0.738301
34	Zr	0.000000	2.088231	3.691505
35	Zr	3.656707	4.182118	6.607444
36	O	1.760037	3.101363	7.409082
37	N	3.098042	3.798377	10.036206
38	C	4.203419	5.534572	13.467896
39	O	4.897278	5.891059	12.589812
40	N	2.592944	3.582481	10.996990
41	O	3.509474	5.176480	14.344948

N_2O formation on ZrO_2 (111) surface --vi

ATOM		X (Angstroms)	Y (Angstroms)	Z (Angstroms)
1	O	0.000000	4.176462	0.000000
2	O	0.000000	0.000000	2.953204
3	O	3.540347	2.145501	5.963610
4	O	3.616922	4.176462	0.000000
5	O	3.616922	0.000000	2.953204
6	O	0.023626	2.057005	5.849231
7	O	5.425383	1.044115	0.000000
8	O	1.808461	3.132346	2.953204
9	O	-1.849141	5.247596	5.875951
10	O	1.808461	1.044115	0.000000
11	O	-1.808461	3.132346	2.953204
12	O	1.862200	5.165867	5.896272
13	O	0.000000	2.088231	1.476602
14	O	3.616922	4.176462	4.429807
15	O	3.636600	-0.094753	7.310382
16	O	3.616922	2.088231	1.476602
17	O	0.000000	4.176462	4.429807
18	O	0.000128	0.039900	7.448671
19	O	1.808461	5.220577	1.476602
20	O	1.808461	1.044115	4.429807
21	O	-1.758282	3.155922	7.346825
22	O	-1.808461	5.220577	1.476602
23	O	5.425383	1.044115	4.429807
24	Zr	0.000000	0.000000	0.738301
25	Zr	3.616922	2.088231	3.691505
26	Zr	-0.072260	4.230550	6.585725
27	Zr	-1.808461	3.132346	0.738301
28	Zr	1.808461	5.220577	3.691505
29	Zr	1.809215	0.995075	6.703707
30	Zr	1.808461	3.132346	0.738301
31	Zr	-1.808461	5.220577	3.691505
32	Zr	5.473254	1.046567	6.618931
33	Zr	3.616922	0.000000	0.738301
34	Zr	0.000000	2.088231	3.691505

35	Zr	3.719202	4.246448	6.626921
36	O	1.433979	3.042036	7.625350
37	N	2.582595	3.280689	8.455253
38	N	2.915342	2.043740	8.805793
39	O	3.927558	1.892459	9.490427

N_2O formation on ZrO_2 (111) surface—TS2

ATOM		X (Angstroms)	Y (Angstroms)	Z (Angstroms)
1	O	0.000000	4.176462	0.000000
2	O	0.000000	0.000000	2.953204
3	O	3.521755	2.162885	5.933362
4	O	3.616922	4.176462	0.000000
5	O	3.616922	0.000000	2.953204
6	O	0.057660	2.115796	5.826939
7	O	5.425383	1.044115	0.000000
8	O	1.808461	3.132346	2.953204
9	O	-1.789195	5.231988	5.907905
10	O	1.808461	1.044115	0.000000
11	O	-1.808461	3.132346	2.953204
12	O	1.828790	5.096759	5.929898
13	O	0.000000	2.088231	1.476602
14	O	3.616922	4.176462	4.429807
15	O	3.669734	0.023878	7.391453
16	O	3.616922	2.088231	1.476602
17	O	0.000000	4.176462	4.429807
18	O	-0.017735	0.056936	7.370748
19	O	1.808461	5.220577	1.476602
20	O	1.808461	1.044115	4.429807
21	O	-1.754269	3.088575	7.366612
22	O	-1.808461	5.220577	1.476602
23	O	5.425383	1.044115	4.429807
24	Zr	0.000000	0.000000	0.738301
25	Zr	3.616922	2.088231	3.691505
26	Zr	-0.020265	4.156560	6.609787
27	Zr	-1.808461	3.132346	0.738301
28	Zr	1.808461	5.220577	3.691505
29	Zr	1.788680	1.052643	6.603330
30	Zr	1.808461	3.132346	0.738301
31	Zr	-1.808461	5.220577	3.691505
32	Zr	5.390249	1.031612	6.662900
33	Zr	3.616922	0.000000	0.738301
34	Zr	0.000000	2.088231	3.691505
35	Zr	3.734737	4.270477	6.631060
36	O	1.447887	2.915151	7.707601
37	N	2.756313	3.593729	8.564701
38	N	2.732491	3.129799	9.700550
39	O	3.532364	3.320586	10.635175

N_2O formation on ZrO_2 (111) surface --vii

ATOM		X (Angstroms)	Y (Angstroms)	Z (Angstroms)
1	O	0.000000	4.176462	0.000000
2	O	0.000000	0.000000	2.953204
3	O	3.622906	2.093556	5.871082
4	O	3.616922	4.176462	0.000000
5	O	3.616922	0.000000	2.953204
6	O	-0.010836	2.089889	5.866212
7	O	5.425383	1.044115	0.000000
8	O	1.808461	3.132346	2.953204
9	O	-1.815686	5.214871	5.865587
10	O	1.808461	1.044115	0.000000
11	O	-1.808461	3.132346	2.953204
12	O	1.813312	5.219827	5.873851
13	O	0.000000	2.088231	1.476602
14	O	3.616922	4.176462	4.429807
15	O	3.607028	-0.006652	7.406239
16	O	3.616922	2.088231	1.476602
17	O	0.000000	4.176462	4.429807
18	O	0.004258	0.010203	7.423627

19	O	1.808461	5.220577	1.476602
20	O	1.808461	1.044115	4.429807
21	O	-1.810907	3.143369	7.428708
22	O	-1.808461	5.220577	1.476602
23	O	5.425383	1.044115	4.429807
24	Zr	0.000000	0.000000	0.738301
25	Zr	3.616922	2.088231	3.691505
26	Zr	-0.005295	4.180605	6.616250
27	Zr	-1.808461	3.132346	0.738301
28	Zr	1.808461	5.220577	3.691505
29	Zr	1.807658	1.051755	6.625543
30	Zr	1.808461	3.132346	0.738301
31	Zr	-1.808461	5.220577	3.691505
32	Zr	5.433828	1.035418	6.613299
33	Zr	3.616922	0.000000	0.738301
34	Zr	0.000000	2.088231	3.691505
35	Zr	3.633309	4.168560	6.608634
36	O	1.801172	3.119064	7.424966
37	N	2.675030	3.462336	11.544730
38	N	3.068487	2.553460	10.979587
39	O	3.476767	1.604954	10.379063

Mechanism involving N_2O-- N_2O adsorption

ATOM		X (Angstroms)	Y (Angstroms)	Z (Angstroms)
1	O	0.000000	4.176462	0.000000
2	O	0.000000	0.000000	2.953204
3	O	3.459694	2.179324	5.932072
4	O	3.616922	4.176462	0.000000
5	O	3.616922	0.000000	2.953204
6	O	0.151725	2.172040	5.919074
7	O	5.425383	1.044115	0.000000
8	O	1.808461	3.132346	2.953204
9	O	-1.814953	5.223866	5.924106
10	O	1.808461	1.044115	0.000000
11	O	-1.808461	3.132346	2.953204
12	O	1.811023	5.045605	5.921254
13	O	0.000000	2.088231	1.476602
14	O	3.616922	4.176462	4.429807
15	O	3.707082	0.044306	7.421287
16	O	3.616922	2.088231	1.476602
17	O	0.000000	4.176462	4.429807
18	O	-0.088551	0.053234	7.435851
19	O	1.808461	5.220577	1.476602
20	O	1.808461	1.044115	4.429807
21	O	-1.800866	3.033538	7.421038
22	O	-1.808461	5.220577	1.476602
23	O	5.425383	1.044115	4.429807
24	Zr	0.000000	0.000000	0.738301
25	Zr	3.616922	2.088231	3.691505
26	Zr	-0.028898	4.188788	6.581162
27	Zr	-1.808461	3.132346	0.738301
28	Zr	1.808461	5.220577	3.691505
29	Zr	1.807051	1.013747	6.575846
30	Zr	1.808461	3.132346	0.738301
31	Zr	-1.808461	5.220577	3.691505
32	Zr	5.425203	1.040809	6.743093
33	Zr	3.616922	0.000000	0.738301
34	Zr	0.000000	2.088231	3.691505
35	Zr	3.643638	4.195425	6.578244
36	O	2.403964	2.554405	10.370138
37	N	1.269293	2.603446	10.742534
38	N	0.183714	2.654457	11.087137

Mechanism involving N_2O-- TS

ATOM	X (au)	Y (au)	Z (au)
O	0.00000000000000	7.89236871082582	0.00000000000000
O	0.00000000000000	0.00000000000000	5.58074743531643
O	6.49309612144016	4.14793531770133	11.33594538194728

O	6.83499179893255	7.89236871082582	0.00000000000000
O	6.83499179893255	0.00000000000000	5.58074743531643
O	0.33055321814701	4.13084714040373	11.25688055612693
O	10.25248770028855	1.97309217723402	0.00000000000000
O	3.41749589946627	5.91927653359180	5.58074743531643
O	-3.42203210577110	9.89950891324362	11.16497591434072
O	3.41749589946627	1.97309217723402	0.00000000000000
O	-3.41749589946627	5.91927653359180	5.58074743531643
O	3.42285445221973	9.47809060487984	11.26888711247578
O	0.00000000000000	3.94618435635777	2.79037371765821
O	6.83499179893255	7.89236871082582	8.37112115297464
O	7.02504688246110	0.11036990408507	14.06536481181362
O	6.83499179893255	3.94618435635777	2.79037371765821
O	0.00000000000000	7.89236871082582	8.37112115297464
O	-0.16324797296052	0.08433731666662	14.11263127471021
O	3.41749589946627	9.86546088805984	2.79037371765821
O	3.41749589946627	1.97309217723402	8.37112115297464
O	-3.42368880031899	5.72832559704780	14.03117801270213
O	-3.41749589946627	9.86546088805984	2.79037371765821
O	10.25248770028855	1.97309217723402	8.37112115297464
Zr	0.00000000000000	0.00000000000000	1.39518685788424
Zr	6.83499179893255	3.94618435635777	6.97593429320067
Zr	-0.11955166758666	7.95387438290644	12.55180438696246
Zr	-3.41749589946627	5.91927653359180	1.39518685788424
Zr	3.41749589946627	9.86546088805984	6.97593429320067
Zr	3.39829635384593	1.82334269372887	12.46542360172249
Zr	3.41749589946627	5.91927653359180	1.39518685788424
Zr	-3.41749589946627	9.86546088805984	6.97593429320067
Zr	10.28411125409711	1.95616793178804	12.67720045966877
Zr	6.83499179893255	0.00000000000000	1.39518685788424
Zr	0.00000000000000	3.94618435635777	6.97593429320067
Zr	7.00923672505211	8.00554354862630	12.38317197200057
O	3.23214600722182	5.46433202523694	16.96722998601209
N	2.68682550189669	5.33433042162291	19.33497467612413
N	1.24431635671640	5.17571086037403	20.97547337539275

Mechanism involving N_2O-- N_2 formation

ATOM		X (Angstroms)	Y (Angstroms)	Z (Angstroms)
1	O	0.000000	4.176462	0.000000
2	O	0.000000	0.000000	2.953204
3	O	3.610135	2.092753	5.866433
4	O	3.616922	4.176462	0.000000
5	O	3.616922	0.000000	2.953204
6	O	0.004416	2.094260	5.867445
7	O	5.425383	1.044115	0.000000
8	O	1.808461	3.132346	2.953204
9	O	-1.807512	5.221039	5.874502
10	O	1.808461	1.044115	0.000000
11	O	-1.808461	3.132346	2.953204
12	O	1.806328	5.215092	5.868626
13	O	0.000000	2.088231	1.476602
14	O	3.616922	4.176462	4.429807
15	O	3.622788	0.009753	7.419393
16	O	3.616922	2.088231	1.476602
17	O	0.000000	4.176462	4.429807
18	O	-0.006309	0.007379	7.417485
19	O	1.808461	5.220577	1.476602
20	O	1.808461	1.044115	4.429807
21	O	-1.811587	3.127580	7.417720
22	O	-1.808461	5.220577	1.476602
23	O	5.425383	1.044115	4.429807
24	Zr	0.000000	0.000000	0.738301
25	Zr	3.616922	2.088231	3.691505
26	Zr	0.003137	4.170501	6.610877
27	Zr	-1.808461	3.132346	0.738301
28	Zr	1.808461	5.220577	3.691505
29	Zr	1.808308	1.044611	6.613169
30	Zr	1.808461	3.132346	0.738301

31	Zr	-1.808461	5.220577	3.691505
32	Zr	5.422716	1.039377	6.623358
33	Zr	3.616922	0.000000	0.738301
34	Zr	0.000000	2.088231	3.691505
35	Zr	3.616936	4.170461	6.615451
36	O	1.808088	3.134804	7.427647
37	N	1.937005	3.089228	11.000683
38	N	0.863843	2.821261	10.953768
